# Genome Analysis of a Zygomycete Fungus *Choanephora cucurbitarum* Elucidates Necrotrophic Features Including Bacterial Genes Related to Plant Colonization

**DOI:** 10.1038/srep40432

**Published:** 2017-01-16

**Authors:** Byoungnam Min, Ji-Hyun Park, Hongjae Park, Hyeon-Dong Shin, In-Geol Choi

**Affiliations:** 1 Department of Biotechnology, College of Life Sciences and Biotechnology, Korea University, Seoul 02841, Korea; 2 Division of Environmental Science and Ecological Engineering, College of Life Sciences and Biotechnology, Korea University, Seoul 02841, Korea

## Abstract

A zygomycete fungus, *Choanephora cucurbitarum* is a plant pathogen that causes blossom rot in cucurbits and other plants. Here we report the genome sequence of *Choanephora cucurbitarum* KUS-F28377 isolated from squash. The assembled genome has a size of 29.1 Mbp and 11,977 protein-coding genes. The genome analysis indicated that *C. cucurbitarum* may employ a plant pathogenic mechanism similar to that of bacterial plant pathogens. The genome contained 11 genes with a Streptomyces subtilisin inhibitor-like domain, which plays an important role in the defense against plant immunity. This domain has been found only in bacterial genomes. Carbohydrate active enzyme analysis detected 312 CAZymes in this genome where carbohydrate esterase family 6, rarely found in dikaryotic fungal genomes, was comparatively enriched. The comparative genome analysis showed that the genes related to sexual communication such as the biosynthesis of β-carotene and trisporic acid were conserved and diverged during the evolution of zygomycete genomes. Overall, these findings will help us to understand how zygomycetes are associated with plants.

The kingdom Fungi has a critical role in the carbon cycle^1^ as well as various interactions with other living organisms. Zygomycetes diverged in the early stage of fungal evolution^2^,^3^ and have various ecological types, which encompass plant and animal pathogens^4^,^5^,^6^,^7^,^8^,^9^, saprobes^10^, and parasites of other fungal species^11^. Mucorales is the largest and best-studied clade in zygomycetes.

The Mucorales fungus *Choanephora cucurbitarum* is a necrotrophic plant pathogen, which causes fruit and blossom rot in cucurbits and other plants, including eggplant, squash, ice plant, okra, pumpkin, and petunia, with varying degrees of severity^12^,^13^,^14^,^15^,^16^,^17^. The infection caused by this fungus is frequently found in warm and humid climates such as tropical and subtropical regions^18^. Several dozen Mucorales genomes have been sequenced, including the opportunistic human pathogenic *Rhizopus delemar*^19^, oleaginous *Mucor circinelloides*^20^, human pathogenic *Lichtheimia corymbifera*^21^, and thermophilic *Rhizomucor miehei*^22^. There are some Mucorales fungi reported as plant pathogen including *Mucor circinelloides* (wound pathogen of noni fruit)^17^, *Rhizopus microsporus* (pathogen of maize, sunflower, and rice)^4^, and *Rhizopus stolonifer* (pathogen of sweet potato)^23^, but none of their genomic features have been analyzed and reported in previous studies.

Fungal plant pathogens are classified by three different lifestyles: necrotrophs, biotrophs, or hemibiotrophs^24^. Fungal pathogens have developed different plant colonization strategies depending on their ecological niches and physiological characteristics. Thus, it is important to understand various forms of plant pathogenicity in a genomic scale, and it can be done by comparing the functional repertoires that shape fungal pathogens’ lifestyles. General approaches for understanding plant pathogenicity include identifying functional domains related to host infection, such as adhesion, detoxification, secondary metabolism, and signal conduction^25^, which can be inferred using Pfam^26^ or Gene Ontology (GO)^27^. In addition, the carbohydrate-active enzyme (CAZyme)^28^ profile can be used to characterize the lifestyles of fungi. Global CAZyme investigations of a kingdom of fungi showed that necrotrophic pathogens have more CAZymes than other ecotypes such as biotrophs and saprobes^29^. Interestingly, biotrophic *Ustilago maydis* has an extremely decreased number of total CAZymes, but instead the gene clusters of secreted virulence factors were found in its genome. This suggests that CAZymes are not the only factors that determine plant pathogenicity in fungi. Secreted effectors have also been investigated because plant pathogenic fungi interact with host cell death machinery via these effectors^30^,^31^.

In pathogen–host interaction, genome evolution via sexual communication is important to host adaptation. β-carotene derivatives, particularly trisporoids, have been identified to be responsible for partner recognition and early sexual differentiation in zygomycetes^32^. Three genes, *tsp1, tsp2*, and *tsp3*, are associated with trisporic acid biosynthesis that encode 4-dihydromethyltrisporate dehydrogenase, 4-dihydrotrisporin dehydrogenase, and carotene oxygenase, respectively^32^. *AcaA* encodes an additional carotene cleavage oxygenase acting on the cleavage product of β-carotene made by *tsp3*-encoded oxygenase in *Phycomyces*^33^. The biosynthesis of β-carotene is well-conserved in Mucorales and is mediated by two genes, *carRA* and *carB*^34^.

Here were report the genome sequence of the plant pathogenic Mucorales fungus *Choanephora cucurbitarum*. We used ten Mucorales and two Glomerales genomes as comparatives: one *Absidia*, two *Lichtheimia*, two *Mucor*, one *Parasitella*, two *Rhizopus*, two *Umbelopsis*, and two *Rhizophagus*. We also used the genomes of three plant pathogenic fungi to explore the unique genomic features in the *C. cucurbitarum* genome in perspective of plant infection strategies; i.e., two hemibiotrophic ascomycetes, *Colletotrichum* species and one biotrophic basidiomycete, *Ustilago maydis*.

## Results and Discussion

### Genome assembly and gene prediction

We sequenced and assembled the genome of *C. cucurbitarum* KUS-F28377 isolated from green squash in Korea. On the basis of high-quality reads (18.8 million reads with 4.3 billion bases), we assembled a genome of 29.1 Mbp with 2,814 scaffolds. The estimated genome size based on the k-mer frequency was 29.2 Mbp, thereby indicating that 99.8% of the entire genome was covered by the assembly (Supplementary Figure S1). The N50 values for the contigs and scaffolds were 24.2 kbp and 27.9 kbp, respectively, and the sequence coverage was 81.3-fold. The read-depth coverage and GC-content profile for each scaffold showed no indication for the sequences of contaminants, symbionts, or parasites in the final assembly (Supplementary Figure S2). The genome sizes of Mucorales varied, ranging from 21.9 Mbp (*U. isabellina* B7317) to 49.6 Mbp (*R. microsporus*), implying that genomic gain and loss have extensively occurred during the evolution of Mucorales genomes. It should be noted that the taxon *R. delemar* has been integrated into *R. arrhizus* as a synonym (http://www.indexfungorum.org/names/NamesRecord.asp?RecordID=162898), but we used *R. delemar* to refer to this species in this study.

**Figure 1:**
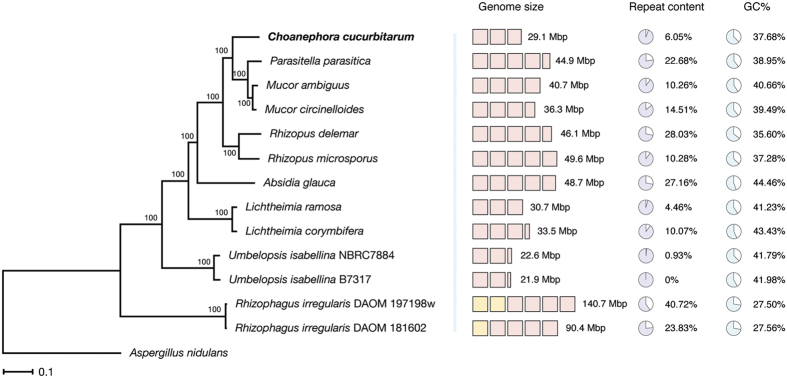
Phylogenetic tree for *C. cucurbitarum* and comparative genomes. The phylogeny was inferred from concatenated single-copy orthologs using RAxML 8.2.7. The bootstrap-based branch supports and the scale bar that represents the mean number of amino acid substitutions per site are shown. *Aspergillus nidulans* was used as an outgroup. Repeat content was estimated using RepeatMasker 4.0.5 with the *de novo* repeat model built by RepeatModeler 1.0.8. The comparative genomes include *Absidia glauca* (GCA_900079185.1), *Lichtheimia corymbifera* (GCA_000723665.1), *Lichtheimia ramosa* (GCA_000945115.1), *Mucor ambiguus* (GCA_000950595.1), *Mucor circinelloides* f. *circinelloides* (GCA_000401635.1), *Parasitella parasitica* (GCA_000938895.1), *Rhizopus delemar* (GCA_000149305.1), *Rhizopus microsporus* (GCA_000325505.1), *Umbelopsis isabellina* B7317 (GCA_000697415.1), *Umbelopsis isabellina* NBRC 7884 (GCA_000534915.1), *Rhizophagus irregularis* DAOM 181602 (GCA_000439145.2), and *Rhizophagus irregularis* DAOM 197198w (GCA_000597685.1). GenBank IDs are indicated in parentheses for each species.

In total, 11,977 protein-coding genes were predicted in the *C. cucurbitarum* genome with an average length of 1,194 nt. The numbers of predicted genes in Mucorales genomes also varied, ranging from 9,082 (*U. isabellina* B7317) to 22,427 (*R. microsporus*). The protein length distribution indicated a slight expansion of short proteins around 100 amino acids (aa) (Supplementary Figure S3). The completeness of the genome assembly and gene predictions were estimated using Benchmarking Universal Single-Copy Orthologs (BUSCO)^35^, which indicated that 97.3% (1,399 entries) of all 1,438 single-copy orthologs were detected in this genome. Table 1 summarizes the genome assembly and gene prediction details and Fig. 1 displays the phylogenetic species tree of *C. cucurbitarum* and the comparatives. The summary of comparatives used in this study is shown in Supplementary Table S1.

### Orthologous and orphan genes

Orthologous gene families were inferred on the basis of the proteomes of *C. cucurbitarum,* ten Mucorales, and two Glomerales genomes. Among the 19,598 inferred gene families, 2,904 (14.82%) families were shared by all 13 genomes (single-member gene families were not counted). The *C. cucurbitarum* genome shared the most gene families with the *M. circinelloides* genome (6,383 families, Supplementary Figure S4). The degree of gene sharing was consistent with the relationships in the phylogenetic tree shown in Fig. 1 (Supplementary Table S2 shows the counts for the shared gene families in all 13 genomes). The number of members per gene family provided little evidence of the whole-genome duplication events, as found in *Rhizopus* genomes^19^, which was consistent with the smaller genome size of the *C. cucurbitarum* genome (Supplementary Figure S4 and Supplementary Table S3).

The *C. cucurbitarum* genome contained 1,326 unique genes (11.11% of overall genes) where their orthologs were absent in any of the comparative genomes used in this study, and they were designated as “orphan genes”. Among 1,326 orphan genes, 1,113 genes belonged to single-member gene families and 213 genes belonged to multi-membered gene families. The second largest gene family for the orphan genes comprised 31 members containing the F-box domain (PF12937), which is related to the ubiquitin proteolysis mechanism^36^. Five orphan genes were annotated with CAZymes: CE4 (acetyl xylan esterase, EC 3.1.1.72), CBM14 (chitin-binding module), GH31 (α-glucosidase, EC 3.2.1.20), GH37 (α,α-trehalase, EC 3.2.1.28), and AA6 (1,4-benzoquinone reductase, EC. 1.6.5.6) modules. These unique CAZymes may determine the host specificity of this organism. Interestingly, the orphan proteins had shorter lengths than those in the overall proteome, where the median lengths of the orphan proteins and all proteins were 107 aa and 318 aa, respectively (Table 2 and Supplementary Figure S5). This bias still needs to be explained. GO annotations showed that the functions of “protein binding,” “oxidation–reduction process,” “ATP binding,” “integral component of membrane,” “serine-type endopeptidase inhibitor activity,” and “metal ion binding” were frequent in these orphan genes.

To identify effector candidates, we predicted 657 genes containing a signal peptide for transport outside of cells. In particular, 97 secreted protein-encoding genes were orphan genes that could potentially function as host-specific effectors, where 84 of these lacked any known functional domain, which is consistent with the current rule for defining effector genes as secreted proteins without known functional domains^30^. The other 13 genes included protease inhibitor domain- and catalase domain-containing genes involved in defense against plant immunity.

Comparative genome analysis showed a various number of orphan genes, ranging from 188 to 8,396, which represents the diverse lifestyles of Mucorales fungi adapting to ecological niches. Orphan genes were often highly duplicated. For instance, *M. ambiguus* genome had an orphan gene family with 69 members. Majority of duplicated orphan genes were not annotated with Pfam or CAZymes. The ortholog groups and the functional annotation of the orphan genes are listed in Supplementary Tables S3 and S4, respectively. Intriguingly, secreted proteins were more likely to be orphan proteins in the *C. cucurbitarum* genome (P value = 0.002 in the chi-square test). This finding suggests that effector genes are under strong selective pressure, which leads to more variation than other genes^30^. This observation was also identified in the comparative genomes except in *R. irregularis* DAOM 181602 and *U. isabellina* NBRC 7884. The orphan genes and statistical tests for secreted orphan proteins are summarized in Table 2 and Supplementary Table S6.

### Pfam domain annotation and bacterial Streptomyces subtilisin inhibitor (SSI)-like domain

**Figure 2:**
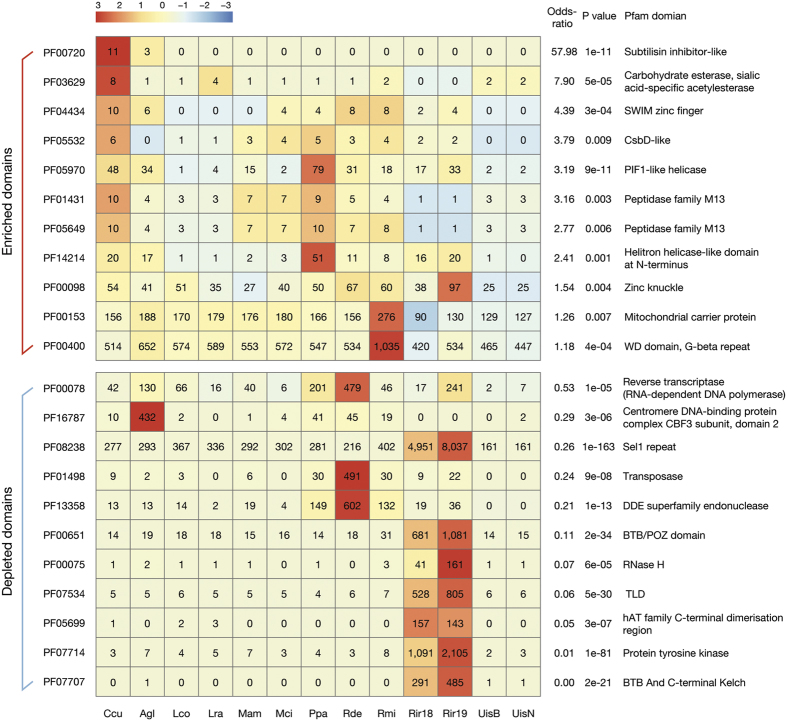
Enriched and depleted Pfam domains in the *C. cucurbitarum* genome compared with other relative genomes. Odds ratios and P values were calculated using Fisher’s exact test. The P value cutoff was 0.05. Scaled values based on row Z-scores were used to fill each cell.

We used Pfam annotations to identify unique or enriched functional domains in the *C. cucurbitarum* genome. We identified 12,767 Pfam domains within 7,936 genes, which was lower than the numbers in the comparative genomes (11,810–28,947 domains, Supplementary Table S7). However, the numbers of unrepeated Pfam entries were similar (3,504 for *C. cucurbitarum* and 3,249–3,676 for the others, Supplementary Figure S6). Indeed, around 65% of the Pfam entries (2,284 entries) were shared by all 13 genomes. In addition, the most frequent Pfam domains were common in all 13 genomes: “protein kinase domain,” “WD domain,” “RNA recognition motif,” “major facilitator superfamily,” and “helicase conserved C-terminal domain” (Supplementary Figure S7). These observations suggest that they share most functions and that small variations determine their lifestyles. The *C. cucurbitarum* genome contained 14 unique, 11 enriched, and 18 depleted Pfam entries (P < 0.01, Fisher’s exact test) compared with the other 12 genomes (Fig. 2).

The most notable enriched domain was the SSI-like domain (PF00720), where 11 copies were present (Fig. 2). It has been reported that only Actinobacteria harbor this domain, which is found mostly in *Streptomyces* spp.^37^. We found that only one other Mucorales fungus, *Absidia glauca,* contains three SSI-like proteins among all the fungal genomes deposited in NCBI. The lengths of the *C. cucurbitarum* SSI-like proteins were relatively short, ranging from 109 aa to 138 aa, and they were encoded by a single exon, except for one containing two exons. Only 6/11 SSI-like genes were supported by mRNA-seq in the hyphal state, which indicates that they might be differently regulated. Eight of the SSI-like proteins had extracellular localizations in the same manner as bacterial homologs (6/8 bacterial SSI-like proteins investigated). The three non-extracellular SSI-like proteins were destined for the endoplasmic reticulum, mitochondria, and cytosol, respectively, thereby suggesting that they may have various roles in cells.

**Figure 3:**
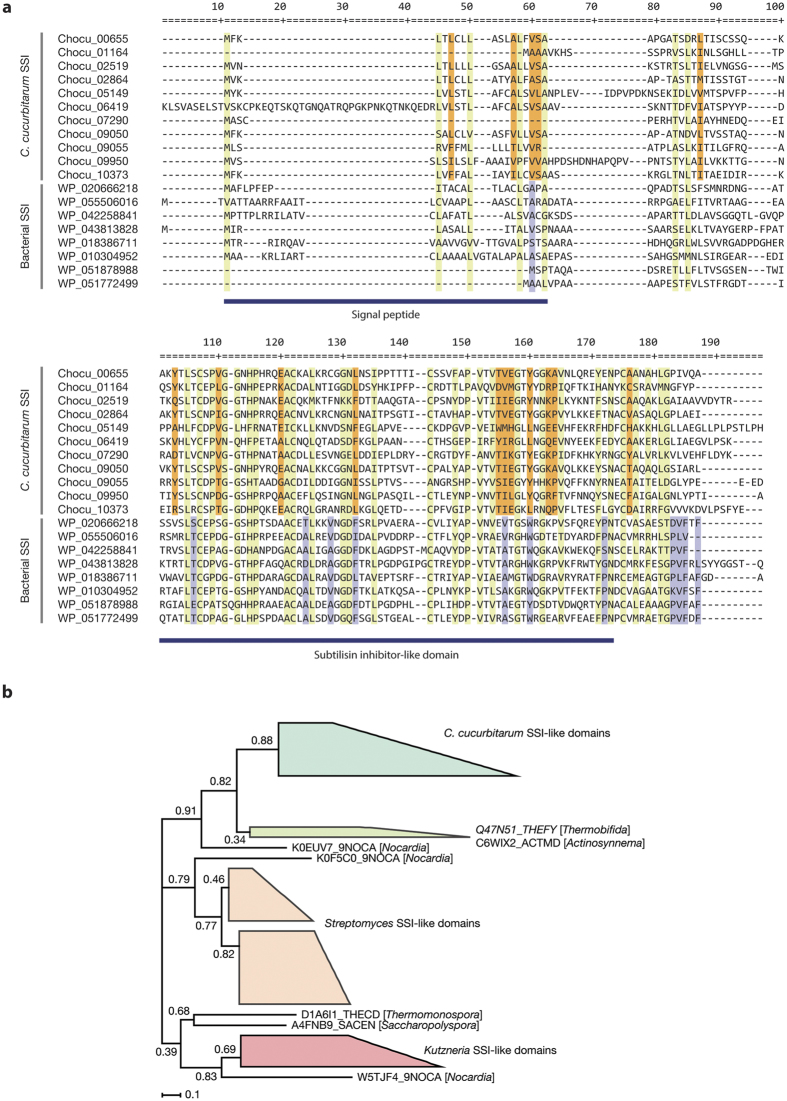
Sequence alignment and gene tree of SSI-like proteins from *C. cucurbitarum* and bacterial genomes. (**a**) Multiple sequence alignment of SSI-like domain-containing protein sequences from *C. cucurbitarum* and bacterial genomes. Conserved positions in all proteins are highlighted in light green, and positions conserved only in *C. cucurbitarum* and bacteria are highlighted in orange and light purple, respectively. GenBank accession numbers are labeled for each sequence: WP_020666218 from *Amycolatopsis nigrescens*, WP_055506016 from *Nonomuraea* sp. NBRC 110462, WP_042258841 from *Nocardia brasiliensis*, WP_043813828 from *Allokutzneria albata*, WP_018386711 from *Streptomyces vitaminophilus*, WP_010304952 from *Saccharopolyspora spinosa*, WP_051878988 from *Streptomyces* sp. NRRL B-24720, and WP_051772499 from *Saccharothrix* sp. NRRL B-16314. (**b**) Gene tree of SSI-like domains built by neighbor-joining method. Clustered nodes were collapsed for clarity. Each node is labeled with UniProt ID with corresponding organism name in brackets. The tree was built using FastTree with the default option.

There were clade-specific conserved positions as well as universally conserved positions in SSI-like protein sequences. We aligned a total of 19 SSI-like protein sequences (11 from *C. cucurbitarum* and eight from bacteria) and identified 35 conserved positions in all 19 sequences. The 16 and 12 positions were conserved only in *C. cucurbitarum* and bacteria, respectively (Fig. 3a). Four cysteine positions that form two disulfide bonds were well conserved in all sequences (positions 107, 122, 144, and 175 in Fig. 3a), which indicates that these disulfide bonds are important for correct structure formation in both groups^37^.

We examined the phylogenetic relatedness between *C. cucurbitarum* and bacterial SSI-like domains by building phylogenetic gene trees. The neighbor-joining tree showed the nestedness among the SSI-like domains of *C. cucurbitarum* and three bacterial species: *Thermobifida, Actinosynnema*, and *Nocardia* (Fig. 3b and Supplementary Figure S8). The maximum-likelihood tree built with the same dataset also supported similar grouping pattern (Supplementary Figure S9). Despite that the bootstrap values of branching nodes were weak in the trees to pinpoint the exact phylogenetic relationship, the tree analysis implied that SSI-like domains of *C. cucurbitarum* have been horizontally transferred. These SSI-like paralogs probably help to neutralize the host’s immunity because subtilisin proteases have functions in pathogen recognition in plants^38^.

### Distributions of CAZymes and the CE6 family

**Figure 4:**
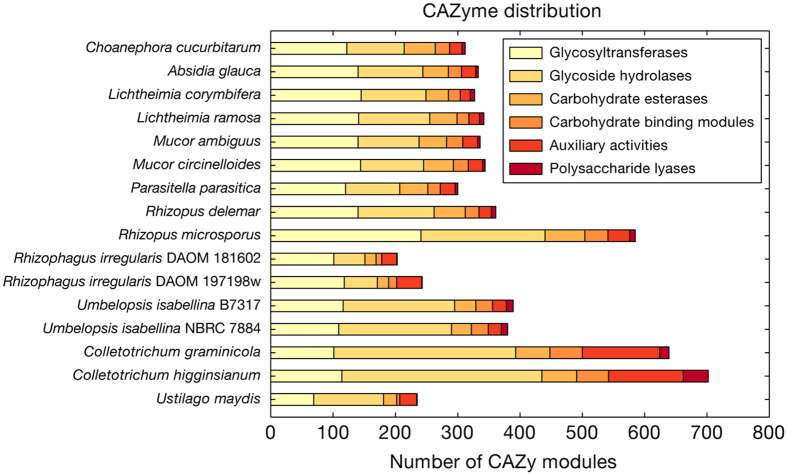
Comparative analysis of CAZymes. The numbers of CAZymes are shown by horizontal bars. The comparative genomes include *Absidia glauca* (GCA_900079185.1), *Lichtheimia corymbifera* (GCA_000723665.1), *Lichtheimia ramosa* (GCA_000945115.1), *Mucor ambiguus* (GCA_000950595.1), *Mucor circinelloides* f. *circinelloides* (GCA_000401635.1), *Parasitella parasitica* (GCA_000938895.1), *Rhizopus delemar* (GCA_000149305.1), *Rhizopus microsporus* (GCA_000325505.1), *Umbelopsis isabellina* B7317 (GCA_000697415.1), *Umbelopsis isabellina* NBRC 7884 (GCA_000534915.1), *Rhizophagus irregularis* DAOM 181602 (GCA_000439145.2), *Rhizophagus irregularis* DAOM 197198w (GCA_000597685.1), *Colletotrichum graminicola* (GCA_000149035.1), *Colletotrichum higginsianum* (GCA_000313795.2), and *Ustilago maydis* (GCA_000328475.2). GenBank IDs are indicated in parentheses for each species.

The *C. cucurbitarum* genome contained fewer CAZymes in total than dikaryotic necrotrophs. This genome contained 312 CAZymes whereas other dikaryotic necrotrophs contained 419–865 CAZymes with an average of 605 as previously reported^29^. This number is lower than the average number of CAZymes in hemibiotrophs (542 CAZymes) and is similar to that found in symbiotic and biotrophic plant pathogens (328 and 313 CAZymes, respectively). Plant pathogens have been known to have more CAZymes than saprobes and biotrophs^29^,^39^ because they need to degrade plant biomass to facilitate their invasion. Thus, this suggests that not all plant pathogens have expanded CAZyme modules and different plant pathogenicity factors, such as secreted effector proteins^40^, would lead to dissimilar CAZyme profiles. Interestingly, a decreased number of total CAZymes was also observed in other Mucorales with an average of 342 CAZymes, except for *R. microsporus* with 585 CAZymes, which might be attributable to whole-genome duplication (Fig. 4 and Supplementary Table S8).

The carbohydrate esterase family 6 (CE6, acetyl xylan esterase) was enriched in the *C. cucurbitarum* genome with seven copies, whereas the other Mucorales had one or two copies of each. This family is only conserved in zygomycetes among all fungal clades^29^, but it is also found in bacterial and plant genomes (http://www.cazy.org/CE6.html). The CE6 family is annotated with “acetyl xylan esterase” and it improves the saccharification of stem lignocellulose by deacetylating xylan^41^. The gene tree of CE6 family indicated that the fungal CE6 genes were more diverged from the common ancestor of plants and the bacterial CE6 family (Supplementary Figure S10).

### Pathogen–host interactions and bacterial virulence genes

The genes involved in pathogen–host interactions (PHIs) were inferred by identifying their homologs in the PHI-base database^42^. Among 11,977 predicted genes, 2,277 (19.01%) genes shared similarity with the PHI genes. In particular, 1,504 genes were related to plant hosts (monocots or eudicots) and there were fewer of these genes than those in the comparative genomes (1,362–2,967 genes, Table 3 and Supplementary Figure S11) and two *Colletotrichum* genomes (2,395 and 2,972 PHI genes in *C. graminicola* and *C. higginsianum*, respectively), which may be attributable to its small genome size. In total, 226 PHI entries were shared by all 16 genomes (*C. cucurbitarum*, ten Mucorales, two Glomerales, two *Colletotrichum*, and *Ustilago*). These common virulence genes determine the core plant-infecting factors, and they are not specific to hosts. Four known effectors were found in the *C. cucurbitarum* genome: two copies of PHI:2703 (*Xanthomonas axonopodis*-derived citrus canker causing effector), one copy of PHI:2347 (grey mould-causing effector), and one copy of PHI:4506 (*Epichloe festucae*-derived effector). Genes involved in host-interactions in *C. cucurbitarum* still need to be fully elucidated, so we expect that many genes were missed in this investigation.

### Sexual communication genes in the Mucorales and Glomerales genomes

**Figure 5:**
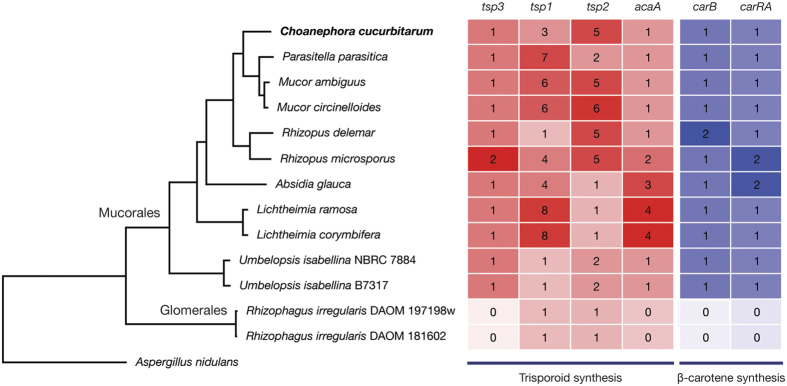
Sexual communication gene distribution in the Mucorales and Glomerales genomes. Four trisporoids synthesis and two β-carotene synthesis-related genes were counted for each genome. Scaled values based on column Z-scores were used to fill each cell. The comparative genomes include *Absidia glauca* (GCA_900079185.1), *Lichtheimia corymbifera* (GCA_000723665.1), *Lichtheimia ramosa* (GCA_000945115.1), *Mucor ambiguus* (GCA_000950595.1), *Mucor circinelloides* f. *circinelloides* (GCA_000401635.1), *Parasitella parasitica* (GCA_000938895.1), *Rhizopus delemar* (GCA_000149305.1), *Rhizopus microsporus* (GCA_000325505.1), *Umbelopsis isabellina* B7317 (GCA_000697415.1), *Umbelopsis isabellina* NBRC 7884 (GCA_000534915.1), *Rhizophagus irregularis* DAOM 181602 (GCA_000439145.2), and *Rhizophagus irregularis* DAOM 197198w (GCA_000597685.1). GenBank IDs are indicated in parentheses for each species.

The analysis of trisporoids and β-carotene biosynthesis-related genes revealed the conservation and divergence of these genes over Mucorales and Glomerales genomes. As shown in Fig. 5, we investigated four genes related to trisporoids biosynthesis (*tsp1, tsp2, tsp3*, and *acaA*) and two genes related to β-carotene biosynthesis (*carB* and *carRA*). *Tsp3*, which encodes carotene oxygenase catalyzing β-carotene cleavage, was well-conserved in the Mucorales genomes as a single copy with an exception of two copies in the *R. microsporus* genome. The *acaA* gene had high sequence similarity with *tsp3* (33% identity and 50% similarity in *Phycomyces* proteins), therefore the ortholog-finding method was applied to differentiate the two genes. Interestingly, *tsp1* (4-dihydromethyltrisporate dehydrogenase) and *tsp2* (4-dihydrotrisporin dehydrogenase) had various degrees of duplications over the genomes ranging from one to eight copies. The phylogenetic tree showed that *tsp1* genes were divided into three groups (Supplementary Figure S12), suggesting that *tsp1* genes were duplicated for functional divergence in contrast to *tsp2* genes (Supplementary Figure S13). Two *R. irregularis* genomes lacked both *tsp3* and *acaA* genes, but contained *tsp1* and *tsp2* genes. This observation suggests the synthesis of signal compounds by non-β-carotene pathway, which is also supported by an absence of *carB* and *carRA* orthologs in these genomes.

## Conclusion

Here were report the genome sequence of *C. cucurbitarum* KUS-F28377, a pathogen of cucurbits and other plants isolated in Korea. We determined the functional profiles of the *C. cucurbitarum* genome and compared them with the relatives and other plant pathogenic fungal genomes to identify the genetic features responsible for the mechanisms of plant pathogenicity unique to this genome. In conclusion, exploring the pathogenicity of *C. cucurbitarum* using genomics can provide crucial insights, which may facilitate the development of disease control strategies.

## Methods and Materials

### Strain isolation and genomic DNA/RNA preparation

The strain *C. cucurbitarum* KUS-F28377 (Korean Agricultural Culture Collection No. 48046, http://www.genebank.go.kr/eng/about/aboutus.jsp) was isolated from *Cucurbita moschata*. Cultures were grown for 10 days on potato dextrose agar (PDA) at 25 °C and the fungal mycelium was harvested. Genomic DNA was extracted by following the protocol provided by Kohler *et al*.^43^.

### Genome sequencing and assembly

In total, 10 million read pairs (300 bp per read and 2.79 billion bases) were generated by Illumina MiSeq paired-end sequencing. The raw reads were trimmed and filtered on the basis of sequence quality (20 Q-score cutoff) and length (20 bp cutoff) using HTQC 1.92.3^44^. A preliminary assembly was generated by ABySS 1.9.0^45^ with the options of k-mer = 41 and insert size = 400 bp. The assembled contigs were used to construct a simulated jumping library with wgsim (https://github.com/lh3/wgsim), which generated 4.5 million read pairs with an insert size of 3 kbp without errors (-e 0 -d 3000 -s 300 -N 5000000 -1 150 -2 150 -r 0 -R 0 -X 0). Finally, 2,814 scaffolds were assembled by Allpaths-LG r47300^46^ with 86.2× sequence coverage. Scaffolds less than 1 kbp were excluded. The potential sequences of contaminants, symbionts, or parasites was checked with Blobology^47^, which plots read-depth coverage calculated by read alignment using Bowtie2^48^ and GC content of each scaffold.

### Gene prediction

Transcriptomic data were obtained by Illumina MiSeq sequencer, which generated 1.2 million paired-end reads with 725 million bases. The raw reads were quality controlled by HTQC, as described above, and assembled with Trinity^49^, which yielded 14,759 contigs (8,895 contigs >500 bp). We generated a gene prediction pool using three tools: Maker 2.31.8^50^, Augustus 3.2.1^51^, and Braker1 (http://exon.gatech.edu/genemark/braker1.html), which produced 32,974 preliminary gene predictions. When gene coding regions are overlapped between two genes, we only kept the more feasible predictions based on the BLASTp bit score against the NCBI *nr* database, which yielded 11,977 genes as the final predicted genes. The proteomes of six relatives were used to support protein homology-based gene prediction in Maker: *L. corymbifera, L. ramosa, M. ambiguus, M. circinelloides, R. microsporus*, and *R. delemar*.

### Genome comparisons

The genomes of ten Mucorales, two Glomerales, and three plant pathogenic dikarya species were used for comparisons with the *C. cucurbitarum* genome. The abbreviations for each organism were used in the figures: Ccu, *Choanephora cucurbitarum*; Agl, *Absidia glauca* CBS 101.48; Lco, *Lichtheimia corymbifera* JMRC:FSU:9682; Lra, *Lichtheimia ramosa*; Mam, *Mucor ambiguus* NBRC 6742; Mci, *Mucor circinelloides* f. *circinelloides* 1006PhL; Ppa, *Parasitella parasitica* CBS 412.66; Rde, *Rhizopus delemar* 99-880; Rmi, *Rhizopus microsporus* ATCC 62417; UisB, *Umbelopsis isabellina* B7317; UisN, *Umbelopsis isabellina* NBRC 7884; Rir18, *Rhizophagus irregularis* DAOM 181602; Rir19, *Rhizophagus irregularis* DAOM 197198w; Cgr, *Colletotrichum graminicola* M1.001; Chi, *Colletotrichum higginsianum* IMI 349063; and Uma, *Ustilago maydis* 521. We predicted genes on two *Umbelopsis* genomes because they were not available in the NCBI database. The gene predictions were performed in the same way that we did to *C. cucurbitarum* genome using RNA-seq reads downloaded from NCBI sequence read archive (accession ID: SRX1003360).

### Pfam domain annotation

InterProScan 5.16–55^52^ was used to annotate the Pfam domains^26^ in the *C. cucurbitarum* and comparative genomes. An enrichment test was performed using Fisher’s exact test embedded in the Python Scipy module^53^. Secreted proteins were predicted using WoLF-PSORT^54^.

### Gene tree of SSI-like proteins

We used curated seed alignment of SSI-like domain sequences downloaded from the Pfam database (http://pfam.xfam.org/family/PF00720), which contained 25 sequences. We extracted domain sequences from the *C. cucurbitarum* SSI-like protein sequences inferred by InterProScan (64–83 aa in length). Using Mafft^55^, we added these sequences into the seed alignment (–seed option). The neighbor-joining and maximum-likelihood gene trees were estimated using FastTree^56^ (with a local bootstrap based on 1,000 resamples) and RAxML^57^ (-f a -x 12345 -p 12345 -# 100 -m PROTGAMMAWAG), respectively.

### Gene families and species tree

Gene families present in *C. cucurbitarum* and the comparative genomes were estimated using OrthoFinder 1.0.6^58^. We extracted a subset of single-copy orthologs and concatenated them to prepare a single data set. We built the phylogenetic tree using MAFFT^55^, Gblocks^59^, ClustalW^60^, and RAxML 8.2.7^57^ (-f a -x 12345 -p 12345 -# 100 -m PROTGAMMAWAG) for multiple sequence alignment, eliminating poorly aligned positions, PHYLIP format conversion, and tree inference, respectively. *Aspergillus nidulans* (http://www.aspgd.org) was used as an outgroup for the rooted tree.

### CAZy prediction

CAZymes^28^ were identified using BLASTp, dbCAN^61^, and Pfam annotations. We assigned a CAZyme annotation when at least two methods yielded the same predictions. For BLASTp, we used the hits with a bit score of >50 and alignment coverage of >25% to remove poorly aligned sequences. An enrichment test was performed using Fisher’s exact test embedded in the Python Scipy module^53^.

### PHI-related genes

Genes potentially involved in PHIs were identified using BLASTp with a bit score cutoff of 50 and a minimum coverage of 25% against the PHI-base database (http://www.phi-base.org/)^42^.

### Sexual communication genes

We retrieved the sexual communication protein sequences from GenBank: *tsp1*, Q01213 (*Mucor mucedo*); *tsp2*, AM937248.1 (*Mucor mucedo*); *tsp3*, CAL64769 (*Blakeslea trispora*); *acaA*, XP_018293784.1 (*Phycomyces blakesleeanus*); *carB*, AAO46892.1 (*Blakeslea trispora*); and *carRA*, AAO46893.1 (*Blakeslea trispora*). We ran BLASTp to the reference sequences and selected the true orthologs using gene families of best hit.

## Additional Information

**Accession codes:** This Whole Genome Shotgun project has been deposited at DDBJ/EMBL/GenBank under the accession LUGH00000000. The version described in this paper is version LUGH01000000.

**How to cite this article:** Min, B. *et al*. Genome Analysis of a Zygomycete Fungus *Choanephora cucurbitarum* Elucidates Necrotrophic Features Including Bacterial Genes Related to Plant Colonization. *Sci. Rep.* **7**, 40432; doi: 10.1038/srep40432 (2017).

**Publisher's note:** Springer Nature remains neutral with regard to jurisdictional claims in published maps and institutional affiliations.

## Supplementary Material

Supplementary Information

Supplementary Dataset 1

## Figures and Tables

**Table 1:** Genome features of *C. cucurbitarum.*

Assembly statistics	
Total contig length (Mbp)	28.89
Total scaffold length (Mbp)	29.10
Average base coverage (fold)	86.2
Number of scaffolds	2,814
N50 contig length (kbp)	24.2
N50 scaffold length (kbp)	27.9
G + C content (overall) (%)	37.41
G + C content (coding region) (%)	41.65
G + C content (non-coding region) (%)	34.38
**Predicted protein-coding genes**	
Predicted genes	11,977
Percent coding (%)	49.17
Average coding sequence size (nt)	1,194
Gene density (genes/Mb)	411.62
Total exons	47,510
Total introns	35,533
Number of introns per gene (median)	3
Number of exons per gene (median)	3
Average exon length (nt)	301.16
Average intron length (nt)	71.39

**Table 2:** Summary of the orphan genes found in *C. cucurbitarum* and comparaitve genomes.

Attributes	Ccu	Agl	Lco	Lra	Mam	Mci	Ppa	Rde	Rmi	Rir18	Rir19	UisB	UisN
Total genes	11,977	14,891	13,557	11,546	11,343	12,227	13,408	17,676	22,427	29,830	29,911	9,082	9,460
Orphan genes	1,326	1,824	1,846	271	426	362	924	2,220	1,595	8,396	5,190	188	525
Percentage	11.1%	12.2%	13.6%	2.3%	3.8%	3%	6.9%	12.6%	7.1%	28.1%	17.4%	2.1%	5.5%
GO terms for orphans	172	401	155	59	52	42	79	176	1,008	748	1,040	24	135
CAZymes for orphans	5	11	4	2	2	1	1	5	27	4	3	2	11
All secreted proteins	657	1,121	1,016	864	749	814	699	799	1,139	895	962	788	738
Secreted proteins for orphans	97	253	198	39	39	36	63	142	133	269	208	44	53
Median protein length in proteome	318	351	348	384	375	361	341	256	313	193	271	387	366
Median protein length for orphans	107	144.5	111	160	109	108.5	134	78	144	104	138	135	119

Organism names corresponding to the abbreviations are listed in the methods section.

**Table 3:** Pathogen–host interaction-related genes.

Host	Origin of pathogenic genes	Ccu	Agl	Lco	Lra	Mam	Mci	Ppa	Rde	Rmi	Rir18	Rir19	UisB	UisN	Cgr	Chi	Uma
Number of genes		11,977	14,891	13,557	11,546	11,343	12,227	13,408	17,463	22,427	29,830	29,911	9,082	9,460	12,020	16,141	6,783
Monocots	Fungi	1,273	1,669	1,602	1,549	1,325	1,410	1,380	1,833	2,503	1,872	2,939	1,122	1,074	1,888	2,293	8,68
	Bacteria	5	8	10	10	11	11	12	6	12	5	5	7	7	7	11	4
	Metazoa	0	0	0	0	0	0	0	0	0	0	0	0	0	0	0	0
Eudicots	Fungi	188	254	271	273	238	220	191	246	376	211	290	249	248	473	636	123
	Bacteria	36	46	40	43	36	39	38	41	70	24	44	30	31	26	31	15
	Metazoa	2	2	2	2	2	2	2	3	6	2	2	2	2	1	1	1
Total		1,504	1,979	1,925	1,877	1,612	1,682	1,623	2,129	2,967	2,114	3,280	1,410	1,362	2,395	2,972	1,011

Organism names corresponding to the abbreviations are listed in the methods section.

## References

[b1] Schimel D. S. Terrestrial ecosystems and the carbon cycle. Glob Chang Biol 1, 77–91 (1995). 10.1111/gcb.12822 25472464

[b2] Koestler T. & Ebersberger I. Zygomycetes, microsporidia, and the evolutionary ancestry of sex determination. Genome Biol Evol 3, 186–194 (2011). 21307298 10.1093/gbe/evr009 PMC3056290

[b3] Corradi N., Hijri M., Fumagalli L. & Sanders I. R. Arbuscular mycorrhizal fungi (Glomeromycota) harbour ancient fungal tubulin genes that resemble those of the chytrids (Chytridiomycota). Fungal Genet. Biol. 41, 1037–1045 (2004). 15465392 10.1016/j.fgb.2004.08.005

[b4] Partida-Martinez L. P. & Hertweck C. Pathogenic fungus harbours endosymbiotic bacteria for toxin production. Nature 437, 884–888 (2005). 16208371 10.1038/nature03997

[b5] Nelson S. C. *Rhizopus Soft Rot of Sweetpotato*. (University of Hawai’i at Manoa, College of Tropical Agriculture and Human Resources, Cooperative Extension Service, 2009).

[b6] Khan Z. U., Ahmad S., Brazda A. & Chandy R. *Mucor circinelloides* as a cause of invasive maxillofacial zygomycosis: an emerging dimorphic pathogen with reduced susceptibility to posaconazole. J. Clin. Microbiol. 47, 1244–1248 (2009). 19171681 10.1128/JCM.02030-08 PMC2668293

[b7] Cheng V. C. . Outbreak of intestinal infection due to *Rhizopus microsporus*. J. Clin. Microbiol. 47, 2834–2843 (2009). 19641069 10.1128/JCM.00908-09 PMC2738128

[b8] Bibashi E. . Wound infection caused by *Lichtheimia ramosa* due to a car accident. Med Mycol Case Rep 2, 7–10 (2012). 24432204 10.1016/j.mmcr.2012.12.001 PMC3885937

[b9] Ribes J. A., Vanover-Sams C. L. & Baker D. J. Zygomycetes in human disease. Clin. Microbiol. Rev. 13, 236–301 (2000). 10756000 10.1128/cmr.13.2.236-301.2000 PMC100153

[b10] Farr D. F., Bills G. F., Chamuris G. P. & Rossman A. Y. Fungi on Plants and Plant Products in the United States (APS press, 1989).

[b11] Schultze K., Schimek C., Wostemeyer J. & Burmester A. Sexuality and parasitism share common regulatory pathways in the fungus *Parasitella parasitica*. Gene 348, 33–44 (2005). 15777660 10.1016/j.gene.2005.01.007

[b12] Kwon J. H. & Jee H. J. Soft Rot of Eggplant (*Solanum melongena*) Caused by *Choanephora cucurbitarum* in Korea. Mycobiology 33, 163–165 (2005). 24049494 10.4489/MYCO.2005.33.3.163 PMC3774878

[b13] Wolf F. A. A squash disease caused by *Choanephora cucurbitarum*. Jour. Agr. Res 8, 319–327 (1917).

[b14] Kagiwada S. . First report of Choanephora rot of ice plant (*Mesembryanthemum crystallinum*) caused by *Choanephora cucurbitarum* in Japan. J. Gen. Plant Pathol. 76, 345–347 (2010).

[b15] Park J. H., Cho S. E., Choi I. Y. & Shin H. D. First report of choanephora rot of okra caused by *Choanephora cucurbitarum* in Korea. J Phytopathol 163, 503–506 (2015).

[b16] Akwaji P. I. . Determination of pathogenicity of *Choanephora cucurbitarum* (Berkeley and ravenel) Thaxt, amongst commonly cultivated vegetables in calabar, cross river state, Nigeria. Int. J. Phytopathol. 3, 7 (2014).

[b17] Holcomb G. First report of petunia blight caused by *Choanephora cucurbitarum* in the United States. Plant Dis. 87, 751–751 (2003). 10.1094/PDIS.2003.87.6.751C 30812878

[b18] Hyde K. D. . One stop shop: backbones trees for important phytopathogenic genera: I. Fungal Divers. 67, 21–125 (2014).

[b19] Ma L. J. . Genomic analysis of the basal lineage fungus *Rhizopus oryzae* reveals a whole-genome duplication. PLoS Genet. 5, e1000549 (2009). 19578406 10.1371/journal.pgen.1000549 PMC2699053

[b20] Wei H. . Genomic, proteomic, and biochemical analyses of oleaginous *Mucor circinelloides*: evaluating its capability in utilizing cellulolytic substrates for lipid production. PLoS One 8, e71068 (2013). 24023719 10.1371/journal.pone.0071068 PMC3762813

[b21] Schwartze V. U. . Gene expansion shapes genome architecture in the human pathogen *Lichtheimia corymbifera*: an evolutionary genomics analysis in the ancient terrestrial mucorales (Mucoromycotina). PLoS Genet. 10, e1004496 (2014). 25121733 10.1371/journal.pgen.1004496 PMC4133162

[b22] Zhou P. . Genome sequence and transcriptome analyses of the thermophilic zygomycete fungus *Rhizomucor miehei*. BMC genomics 15, 294 (2014). 24746234 10.1186/1471-2164-15-294 PMC4023604

[b23] Srivastava D. & Walker J. Mechanisms of infection of sweet potato roots by *Rhizopus*-*stolonifer*. Phytopathology 49, 400–406 (1959).

[b24] Glazebrook J. Contrasting mechanisms of defense against biotrophic and necrotrophic pathogens. Annu Rev Phytopathol 43, 205–227 (2005). 16078883 10.1146/annurev.phyto.43.040204.135923

[b25] Guo L. . Genome and transcriptome analysis of the fungal pathogen *Fusarium oxysporum* f. sp. *cubense* causing banana vascular wilt disease. PLoS One 9, e95543 (2014). 24743270 10.1371/journal.pone.0095543 PMC3990668

[b26] Finn R. D. . Pfam: the protein families database. Nucleic Acids Res. 42, D222–230 (2014). 24288371 10.1093/nar/gkt1223 PMC3965110

[b27] Ashburner M. . Gene ontology: tool for the unification of biology. The Gene Ontology Consortium. Nat. Genet. 25, 25–29 (2000). 10802651 10.1038/75556 PMC3037419

[b28] Lombard V., Golaconda Ramulu H., Drula E., Coutinho P. M. & Henrissat B. The carbohydrate-active enzymes database (CAZy) in 2013. Nucleic Acids Res. 42, D490–495 (2014). 24270786 10.1093/nar/gkt1178 PMC3965031

[b29] Zhao Z., Liu H., Wang C. & Xu J. R. Correction: Comparative analysis of fungal genomes reveals different plant cell wall degrading capacity in fungi. BMC genomics 15, 6 (2014). 24422981 10.1186/1471-2164-15-6 PMC3893384

[b30] Lo Presti L. . Fungal effectors and plant susceptibility. Annu Rev Plant Biol 66, 513–545 (2015). 25923844 10.1146/annurev-arplant-043014-114623

[b31] Verma S. . Draft genome sequencing and secretome analysis of fungal phytopathogen *Ascochyta rabiei* provides insight into the necrotrophic effector repertoire. Sci Rep 6, 24638 (2016). 27091329 10.1038/srep24638 PMC4835772

[b32] Schimek C. & Wostemeyer J. Carotene derivatives in sexual communication of zygomycete fungi. Phytochemistry 70, 1867–1875 (2009). 19665150 10.1016/j.phytochem.2009.07.014

[b33] Medina H. R., Cerda-Olmedo E. & Al-Babili S. Cleavage oxygenases for the biosynthesis of trisporoids and other apocarotenoids in *Phycomyces*. Mol. Microbiol. 82, 199–208 (2011). 21854466 10.1111/j.1365-2958.2011.07805.x

[b34] Catalina S., Mahdi S. & Arturo P. E. Biotechnology of Fungal Genes 21–52 (Science Publishers, 2012).

[b35] Simao F. A., Waterhouse R. M., Ioannidis P., Kriventseva E. V. & Zdobnov E. M. BUSCO: assessing genome assembly and annotation completeness with single-copy orthologs. Bioinformatics 31, 3210–3212 (2015). 26059717 10.1093/bioinformatics/btv351

[b36] Bai C. . SKP1 connects cell cycle regulators to the ubiquitin proteolysis machinery through a novel motif, the F-box. Cell 86, 263–274 (1996). 8706131 10.1016/s0092-8674(00)80098-7

[b37] Taguchi S., Kojima S., Terabe M., Miura K. & Momose H. Comparative studies on the primary structures and inhibitory properties of subtilisin-trypsin inhibitors from Streptomyces. Eur. J. Biochem. 220, 911–918 (1994). 8143745 10.1111/j.1432-1033.1994.tb18694.x

[b38] Figueiredo A., Monteiro F. & Sebastiana M. Subtilisin-like proteases in plant-pathogen recognition and immune priming: a perspective. Front Plant Sci 5, 739 (2014). 25566306 10.3389/fpls.2014.00739 PMC4271589

[b39] O’Connell R. J. . Lifestyle transitions in plant pathogenic *Colletotrichum* fungi deciphered by genome and transcriptome analyses. Nat. Genet. 44, 1060–1065 (2012). 22885923 10.1038/ng.2372 PMC9754331

[b40] Kamper J. . Insights from the genome of the biotrophic fungal plant pathogen *Ustilago maydis*. Nature 444, 97–101 (2006). 17080091 10.1038/nature05248

[b41] Pawar P. M. . Expression of fungal acetyl xylan esterase in *Arabidopsis thaliana* improves saccharification of stem lignocellulose. Plant Biotechnol. J. 14, 387–397 (2016). 25960248 10.1111/pbi.12393 PMC11389080

[b42] Urban M. . The Pathogen-Host Interactions database (PHI-base): additions and future developments. Nucleic Acids Res. 43, D645–655 (2015). 25414340 10.1093/nar/gku1165 PMC4383963

[b43] Kohler A., Murat C. & Costa M. High quality genomic DNA extraction using CTAB and Qiagen genomic-tip. Available at: http://1000.fungalgenomes.org/home/wp-content/uploads/2013/02/genomicDNAProtocol-AK0511.pdf (Accessed: 10th August 2016) (2011).

[b44] Yang X. . HTQC: a fast quality control toolkit for Illumina sequencing data. BMC Bioinformatics 14, 33 (2013). 23363224 10.1186/1471-2105-14-33 PMC3571943

[b45] Simpson J. T. . ABySS: a parallel assembler for short read sequence data. Genome Res. 19, 1117–1123 (2009). 19251739 10.1101/gr.089532.108 PMC2694472

[b46] Gnerre S. . High-quality draft assemblies of mammalian genomes from massively parallel sequence data. Proc. Natl. Acad. Sci. USA 108, 1513–1518 (2011). 21187386 10.1073/pnas.1017351108 PMC3029755

[b47] Kumar S., Jones M., Koutsovoulos G., Clarke M. & Blaxter M. Blobology: exploring raw genome data for contaminants, symbionts and parasites using taxon-annotated GC-coverage plots. Front Genet 4, 237 (2013). 24348509 10.3389/fgene.2013.00237 PMC3843372

[b48] Langmead B. & Salzberg S. L. Fast gapped-read alignment with Bowtie 2. Nat. Methods 9, 357–359 (2012). 22388286 10.1038/nmeth.1923 PMC3322381

[b49] Grabherr M. G. . Full-length transcriptome assembly from RNA-Seq data without a reference genome. Nat. Biotechnol. 29, 644–652 (2011). 21572440 10.1038/nbt.1883 PMC3571712

[b50] Holt C. & Yandell M. MAKER2: an annotation pipeline and genome-database management tool for second-generation genome projects. BMC Bioinformatics 12, 491 (2011). 22192575 10.1186/1471-2105-12-491 PMC3280279

[b51] Stanke M. & Morgenstern B. AUGUSTUS: a web server for gene prediction in eukaryotes that allows user-defined constraints. Nucleic Acids Res. 33, W465–467 (2005). 15980513 10.1093/nar/gki458 PMC1160219

[b52] Jones P. . InterProScan 5: genome-scale protein function classification. Bioinformatics 30, 1236–1240 (2014). 24451626 10.1093/bioinformatics/btu031 PMC3998142

[b53] Jones E., Oliphant T. & Peterson P. SciPy: Open Source Scientific Tools for Python. Available at: http://www.scipy.org (Accessed: 10th August 2016) (2001).

[b54] Horton P. . WoLF PSORT: protein localization predictor. Nucleic Acids Res. 35, W585–587 (2007). 17517783 10.1093/nar/gkm259 PMC1933216

[b55] Katoh K., Misawa K., Kuma K. & Miyata T. MAFFT: a novel method for rapid multiple sequence alignment based on fast Fourier transform. Nucleic Acids Res. 30, 3059–3066 (2002). 12136088 10.1093/nar/gkf436 PMC135756

[b56] Price M. N., Dehal P. S. & Arkin A. P. FastTree: computing large minimum evolution trees with profiles instead of a distance matrix. Mol. Biol. Evol. 26, 1641–1650 (2009). 19377059 10.1093/molbev/msp077 PMC2693737

[b57] Stamatakis A. RAxML version 8: a tool for phylogenetic analysis and post-analysis of large phylogenies. Bioinformatics 30, 1312–1313 (2014). 24451623 10.1093/bioinformatics/btu033 PMC3998144

[b58] Emms D. M. & Kelly S. OrthoFinder: solving fundamental biases in whole genome comparisons dramatically improves orthogroup inference accuracy. Genome Biol. 16, 157 (2015). 26243257 10.1186/s13059-015-0721-2 PMC4531804

[b59] Talavera G. & Castresana J. Improvement of phylogenies after removing divergent and ambiguously aligned blocks from protein sequence alignments. Syst. Biol. 56, 564–577 (2007). 17654362 10.1080/10635150701472164

[b60] Thompson J. D., Higgins D. G. & Gibson T. J. CLUSTAL W: improving the sensitivity of progressive multiple sequence alignment through sequence weighting, position-specific gap penalties and weight matrix choice. Nucleic Acids Res. 22, 4673–4680 (1994). 7984417 10.1093/nar/22.22.4673 PMC308517

[b61] Yin Y. . dbCAN: a web resource for automated carbohydrate-active enzyme annotation. Nucleic Acids Res. 40, W445–451 (2012). 22645317 10.1093/nar/gks479 PMC3394287

