# Supplementary material for: Genome Analysis of a Zygomycete Fungus Choanephora cucurbitarum Elucidates Necrotrophic Features Including Bacterial Genes Related to Plant Colonization
Source: Sci Rep. 2017 Jan 16;7:40432. doi: 10.1038/srep40432 (PMC5238444; doi:10.1038/srep40432)
Supplement: Supplementary Information [file srep40432-s1.pdf]

Genome Analysis of a Zygomycete Fungus *Choanephora cucurbitarum* Elucidates Necrotrophic Features Including Bacterial Genes Related to Plant Colonization

Byoungnam Min, Ji-Hyun Park, Hongjae Park, Hyeon-Dong Shin[*], and In-Geol Choi[*]

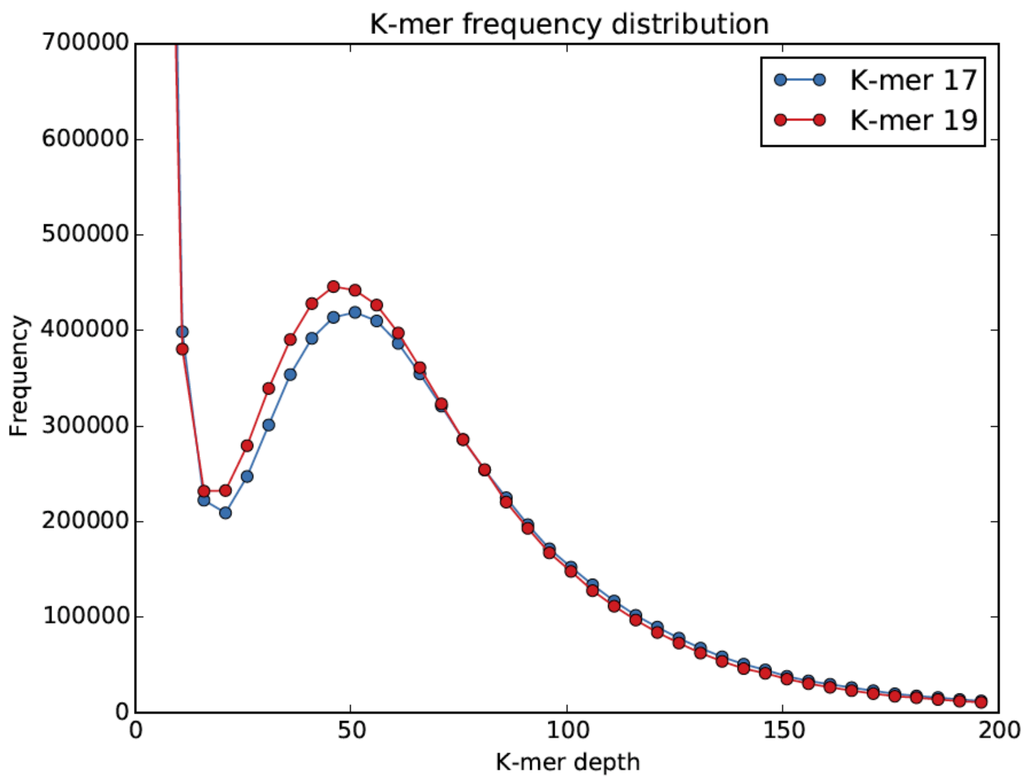

Supplementary Figure S1. K-mer distribution of sequenced reads from *C. cucurbitarum* genomic DNA. Different k-mer lengths are indicated by the colors.

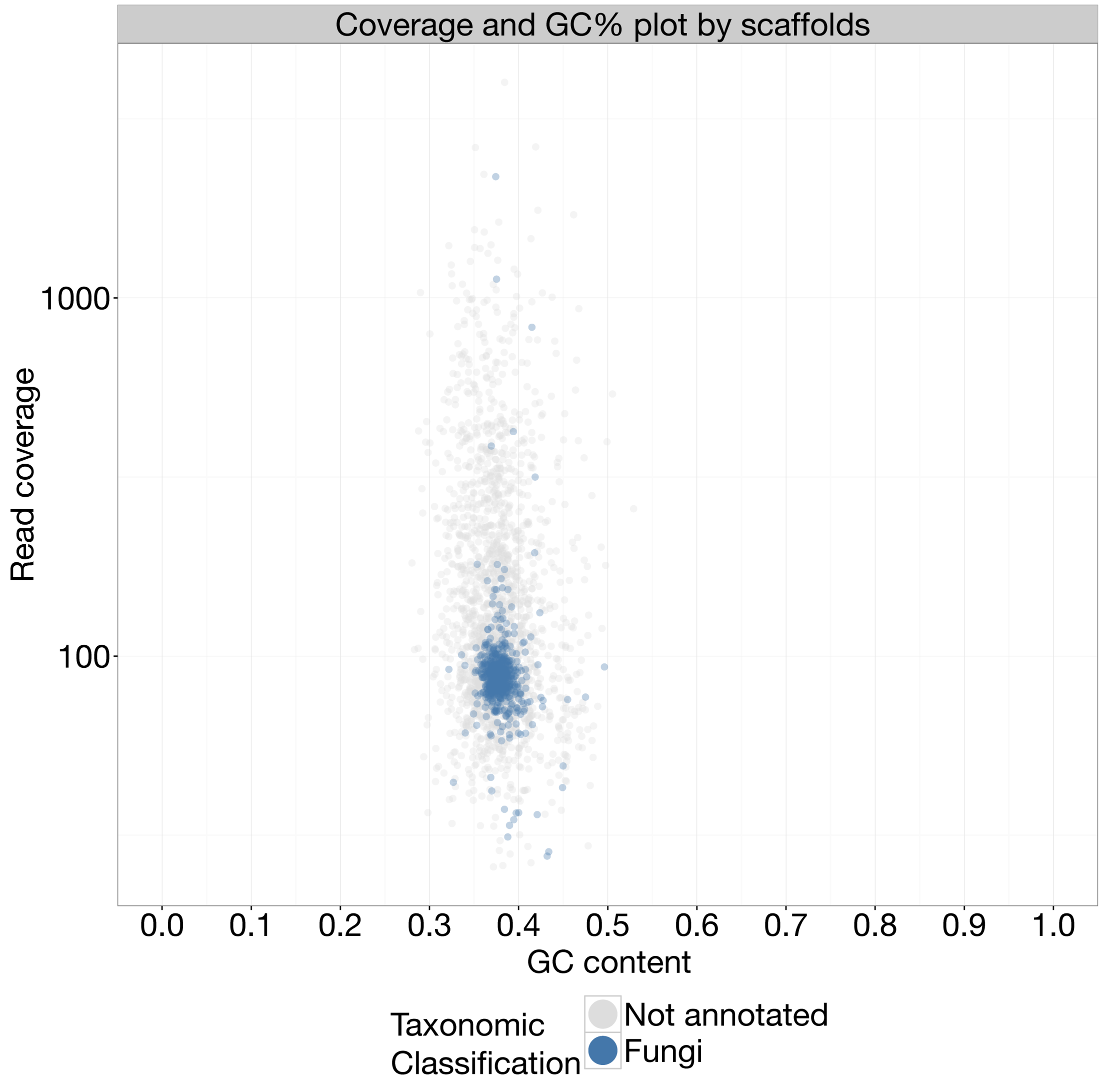

Supplementary Figure S2. Read-depth coverage and GC content plot of *C. cucurbitarum* genome assembly. Each point indicates a scaffold and is labeled by the colors based on taxonomy.

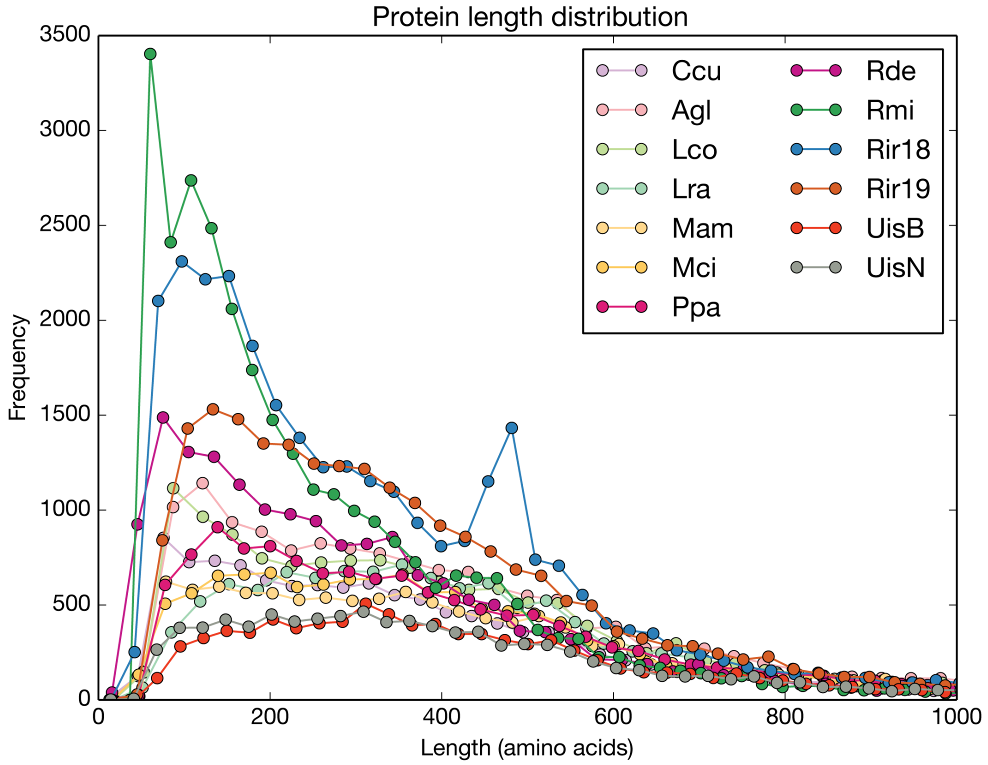

Supplementary Figure S3. Protein length distribution in the *C. cucurbitarum* and comparative genomes. The bin value used for calculating the histogram was 200. Organism names corresponding to the abbreviations in the legend are listed in the methods section. The region beyond 1,000 aa is not shown.

a

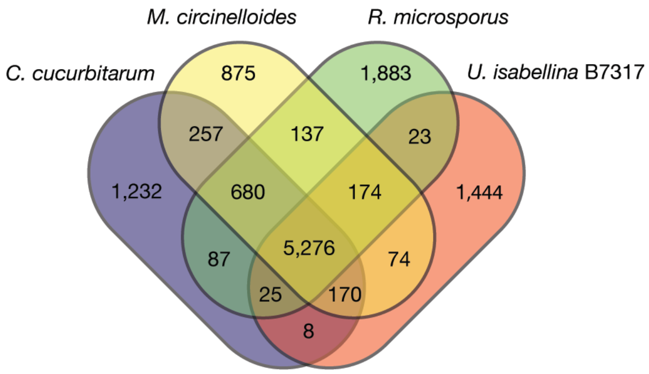

b

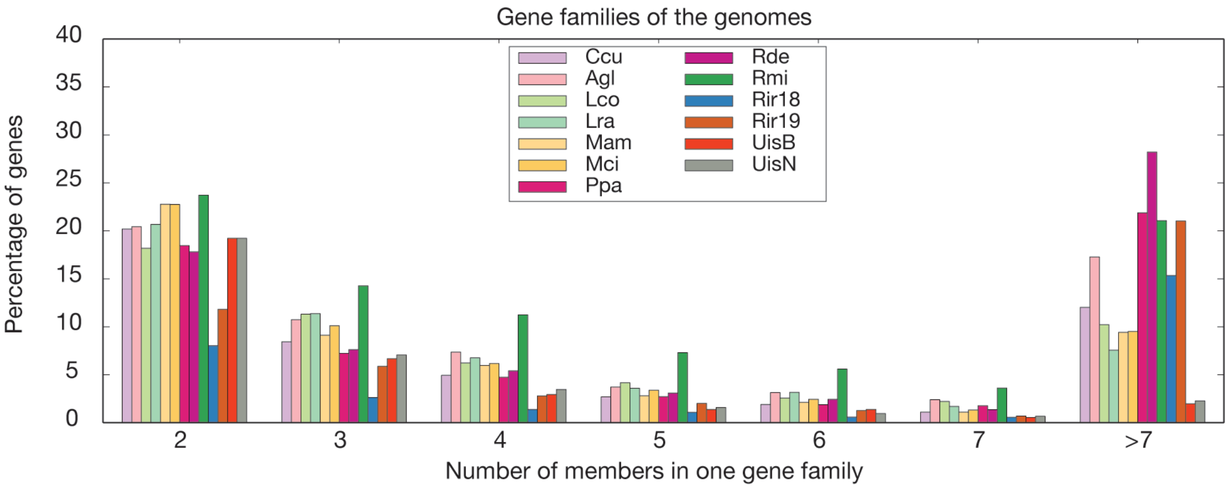

Supplementary Figure S4. Gene families of *C. cucurbitarum* and comparatives. (a) Venn diagram of orthologous gene families among *C. cucurbitarum* and other three genomes. The number of gene families is labeled in each set (the number of genes is not shown in the diagram). (b) The frequencies of gene members in one family. Organism names corresponding to the abbreviations in the legend are listed in the methods section.

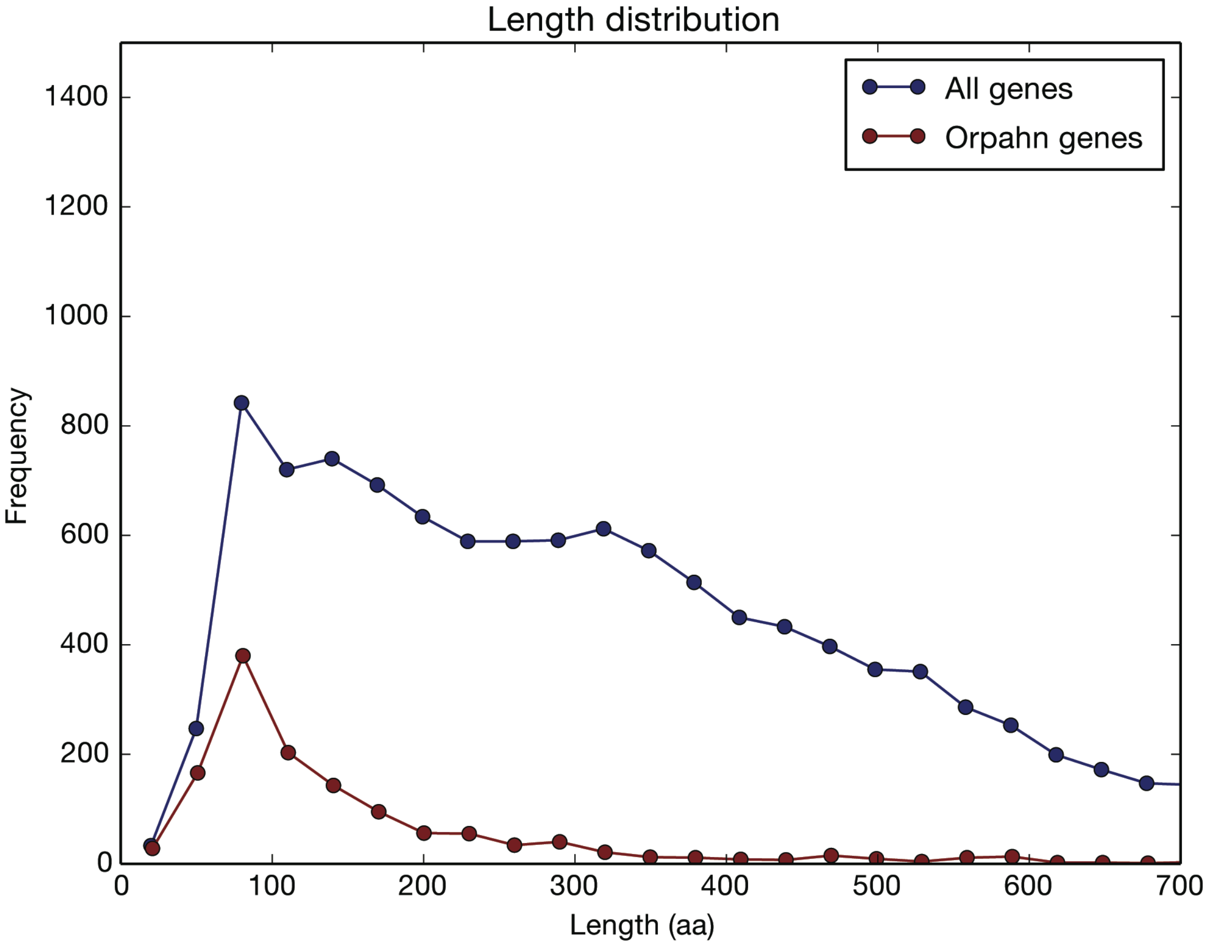

Supplementary Figure S5. Protein length distribution of all predicted genes and the orphan genes whose orthologs are not found in the comparative genomes.

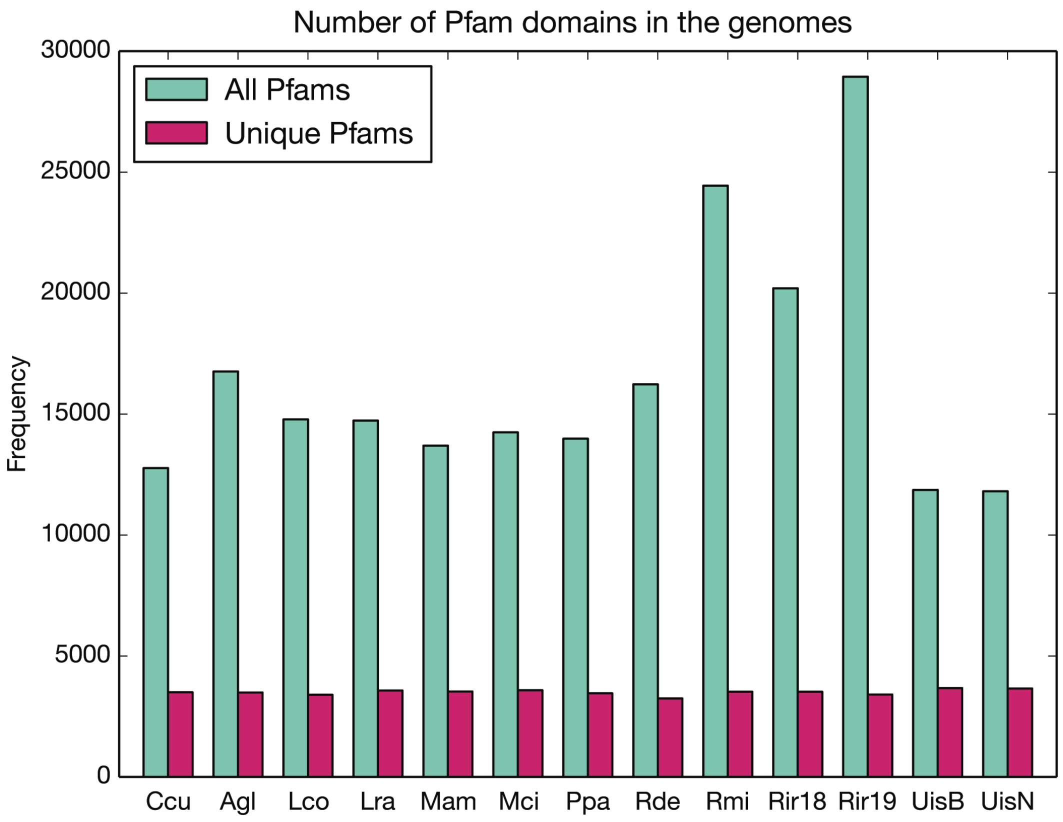

Supplementary Figure S6. Numbers of Pfam domains predicted in the *C. cucurbitarum* and comparative genomes. Organism names corresponding to the abbreviations in the legend are listed in the methods section.

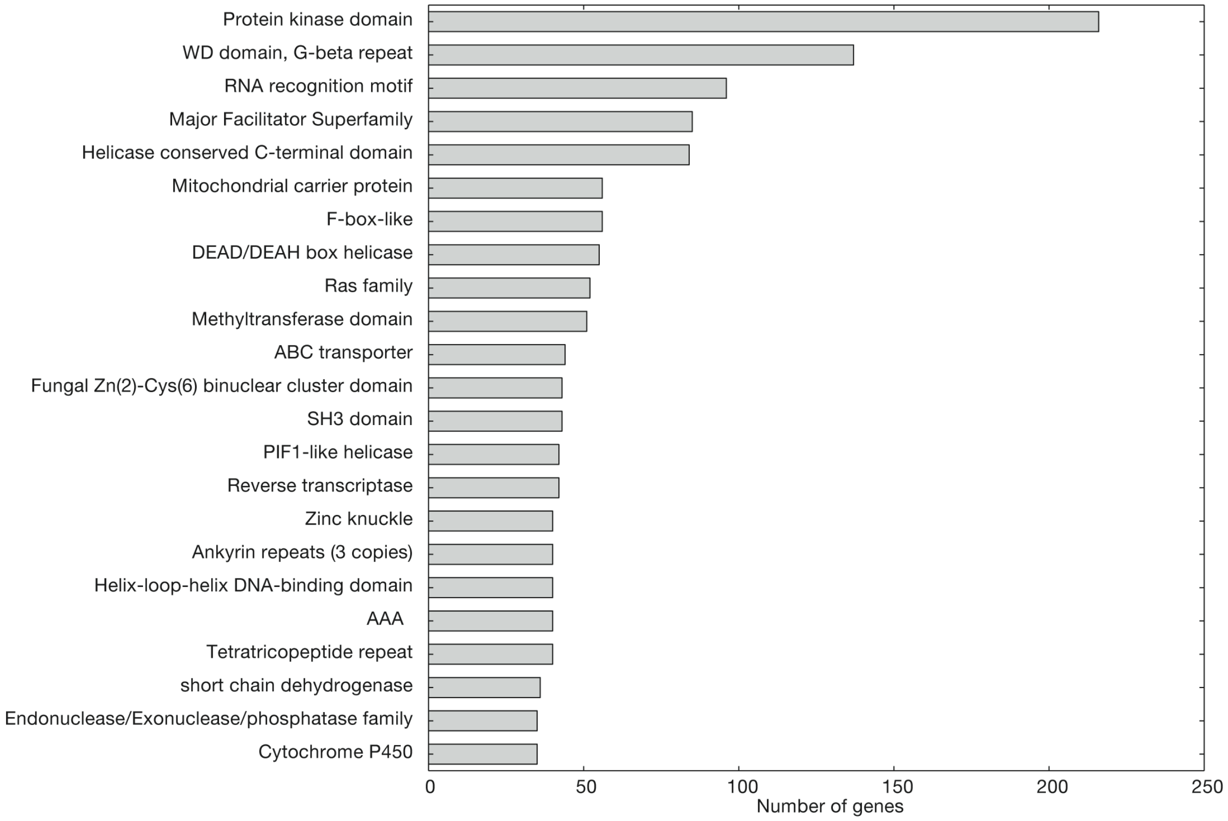

Supplementary Figure S7. Frequent Pfam domains found in the *C. cucurbitarum* genome.

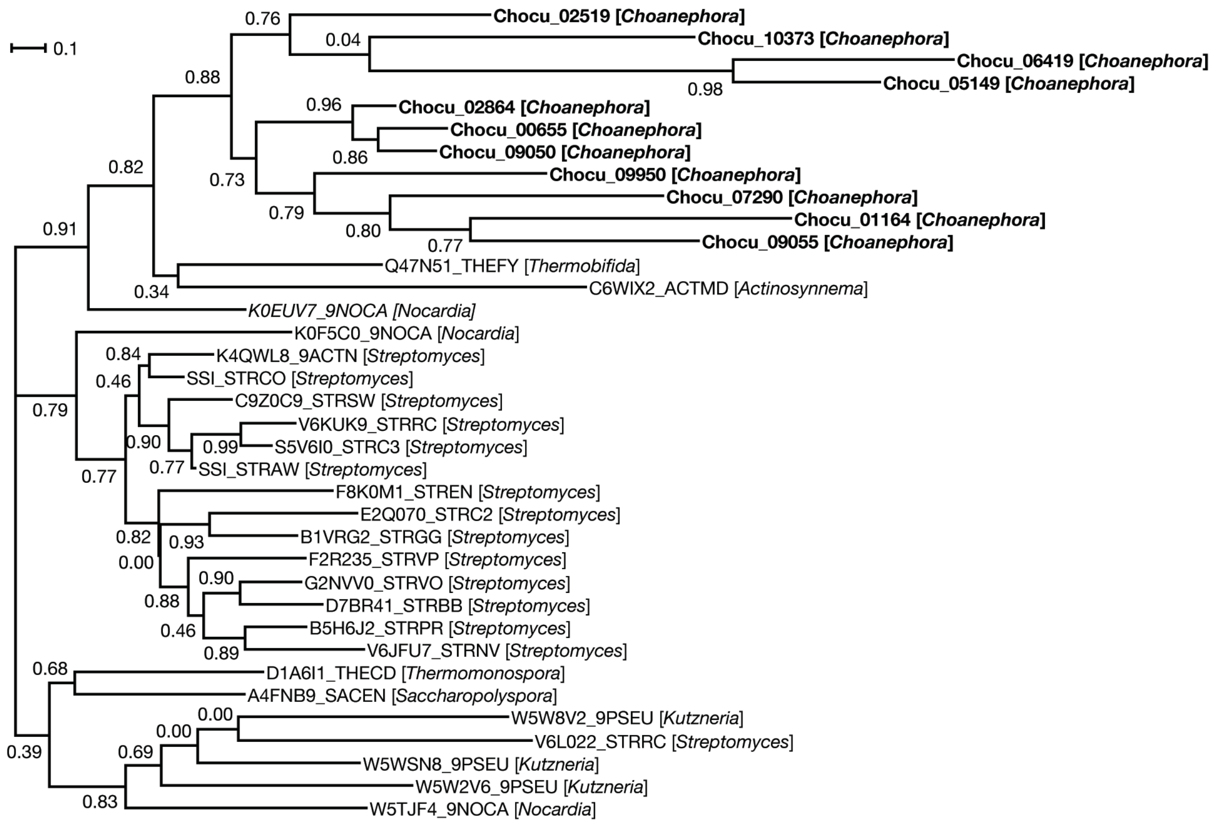

Supplementary Figure S8. Gene tree of SSI-like domains (Neighbor-joining method). Each node is labeled with UniProt ID with corresponding organism name in brackets. The nodes representing *C. cucurbitarum* proteins are in bold. The bootstrap-based branch supports and the scale bar that represents the mean number of amino acid substitutions per site are shown. The tree was built using FastTree with the default option.

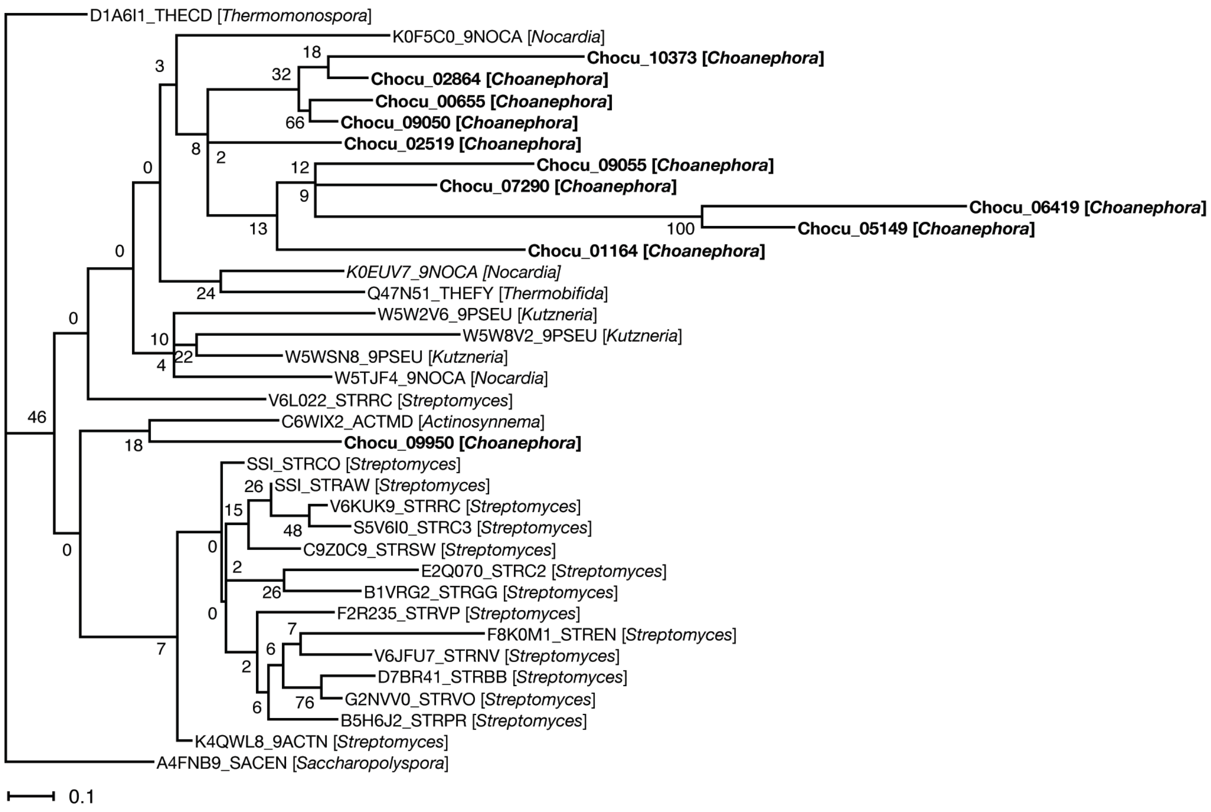

Supplementary Figure S9. Gene tree of SSI-like domains (Maximum-likelihood method). Each node is labeled with UniProt ID with corresponding organism name in brackets. The nodes representing *C. cucurbitarum* proteins are in bold. The bootstrap-based branch supports and the scale bar that represents the mean number of amino acid substitutions per site are shown. The tree was built using RAxML 8.2.7 (-f a -x 12345 -p 12345 -# 100 -m PROTGAMMAWAG).

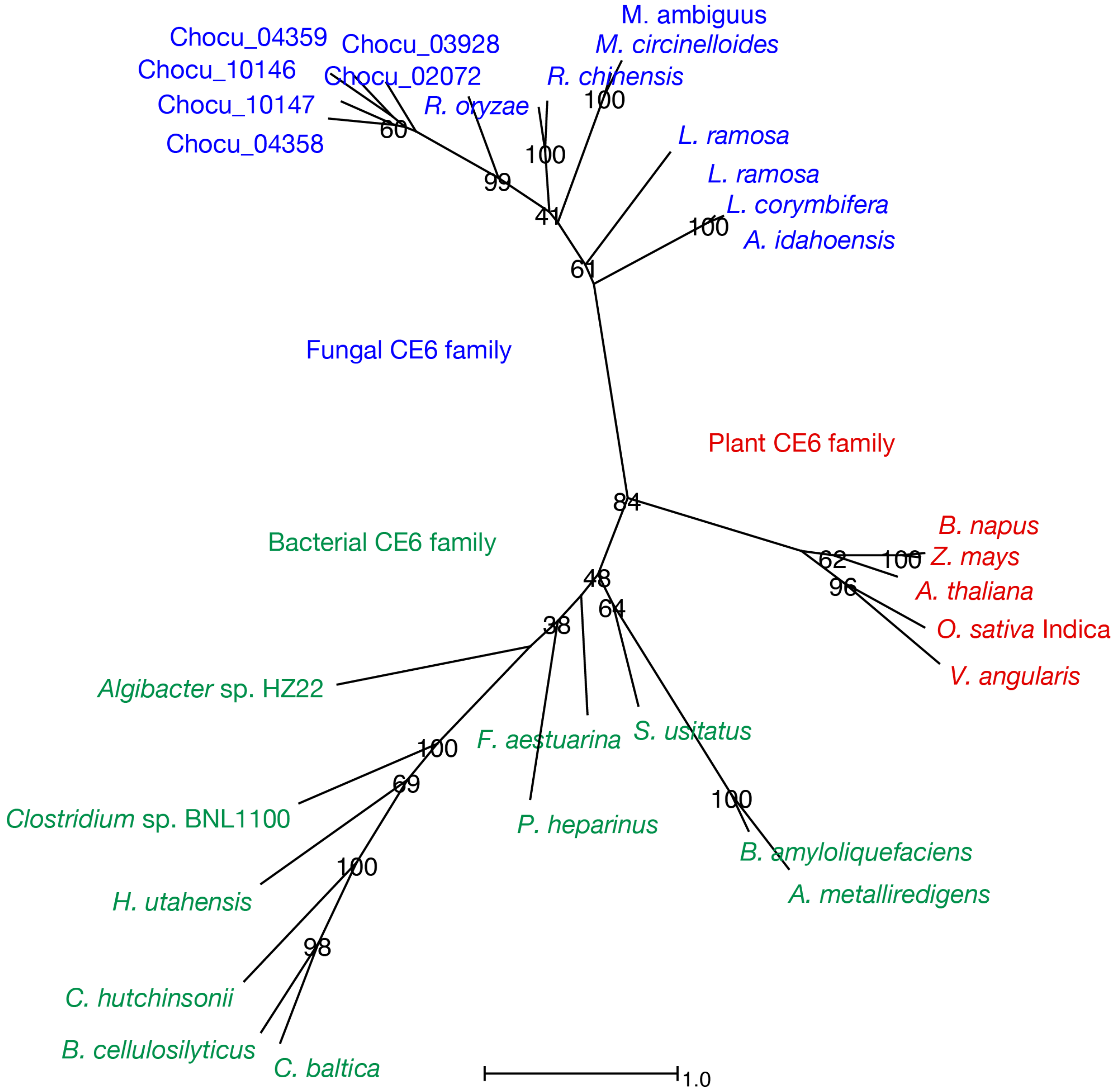

Supplementary Figure S10. Gene tree of CE6 families among multiple clades. Species names containing each CE6 gene are labeled at the nodes with the clade-specific colors. The bootstrap-based branch supports and the scale bar that represents the mean number of amino acid substitutions per site are shown. The tree was built using RAxML 8.2.7 (-f a -x 12345 -p 12345 -# 1000 -m PROTGAMMAWAG).

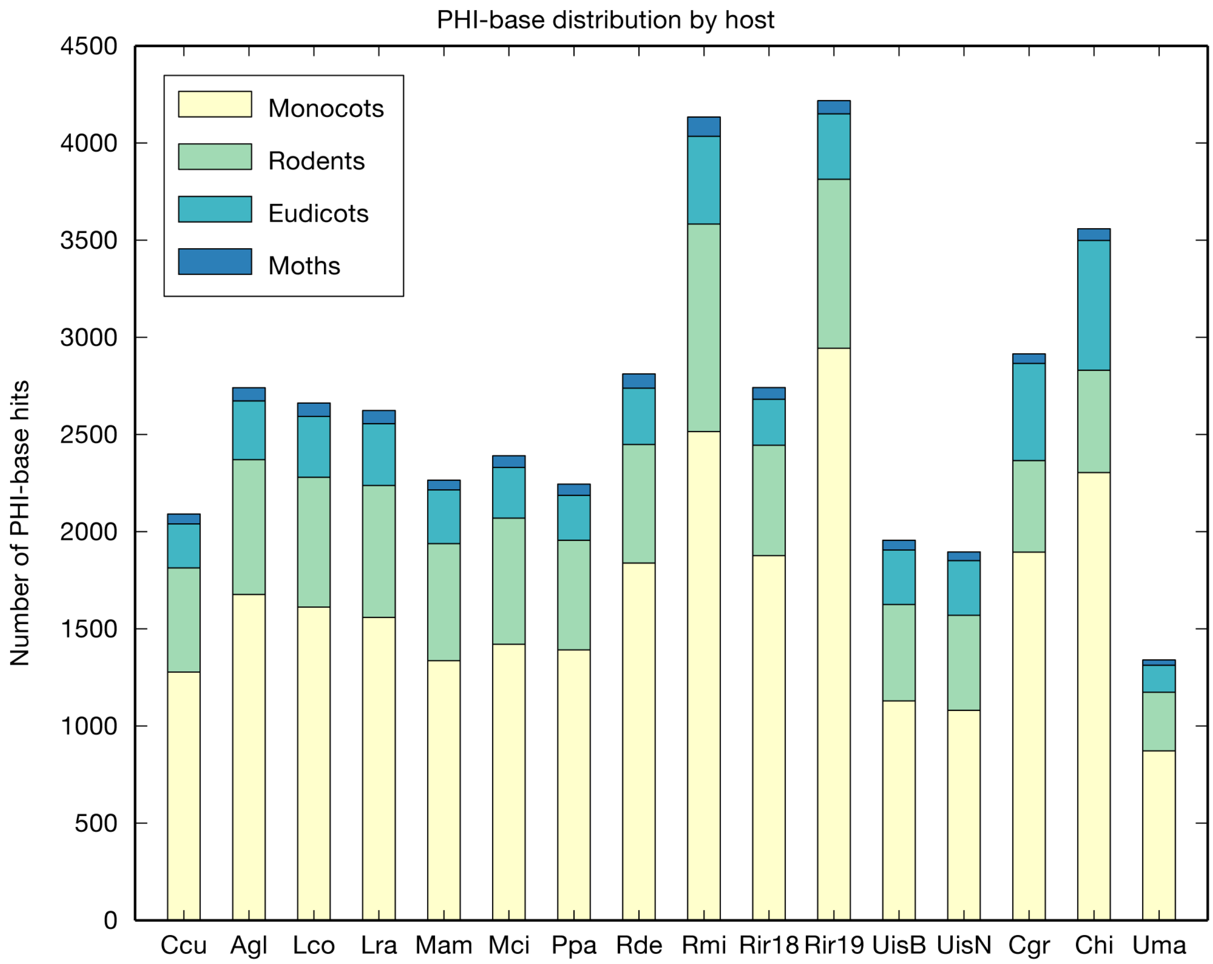

Supplementary Figure S11. Genes related to pathogen–host interactions. The numbers of BLASTp hits against the PHI-base database are shown. Each bar is split by host. Organism names corresponding to the abbreviations in the X-axis are listed in the methods section.

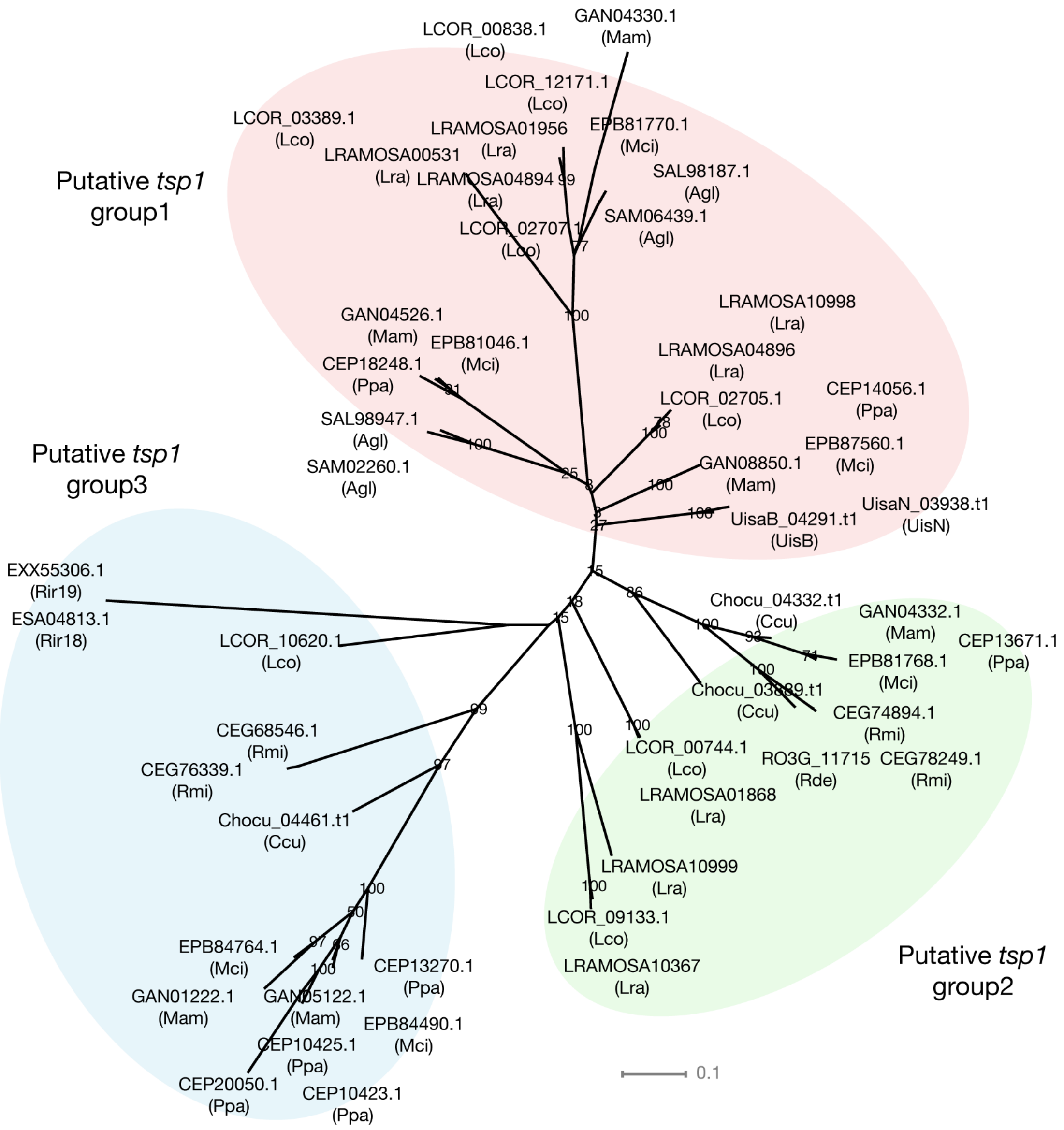

Supplementary Figure S12. Gene tree of *tsp1*. The labels at nodes indicate the GenBank IDs except for *C. cucurbitarum* and two *Umbelopsis* where the locus tags were used. The abbreviations of organisms are shown in parentheses, and the corresponding organism names are listed in the methods section. The bootstrap-based branch supports and the scale bar that represents the mean number of amino acid substitutions per site are shown. The tree was built using RAxML 8.2.7 (-f a -x 12345 -p 12345 -# 100 -m PROTGAMMAWAG).

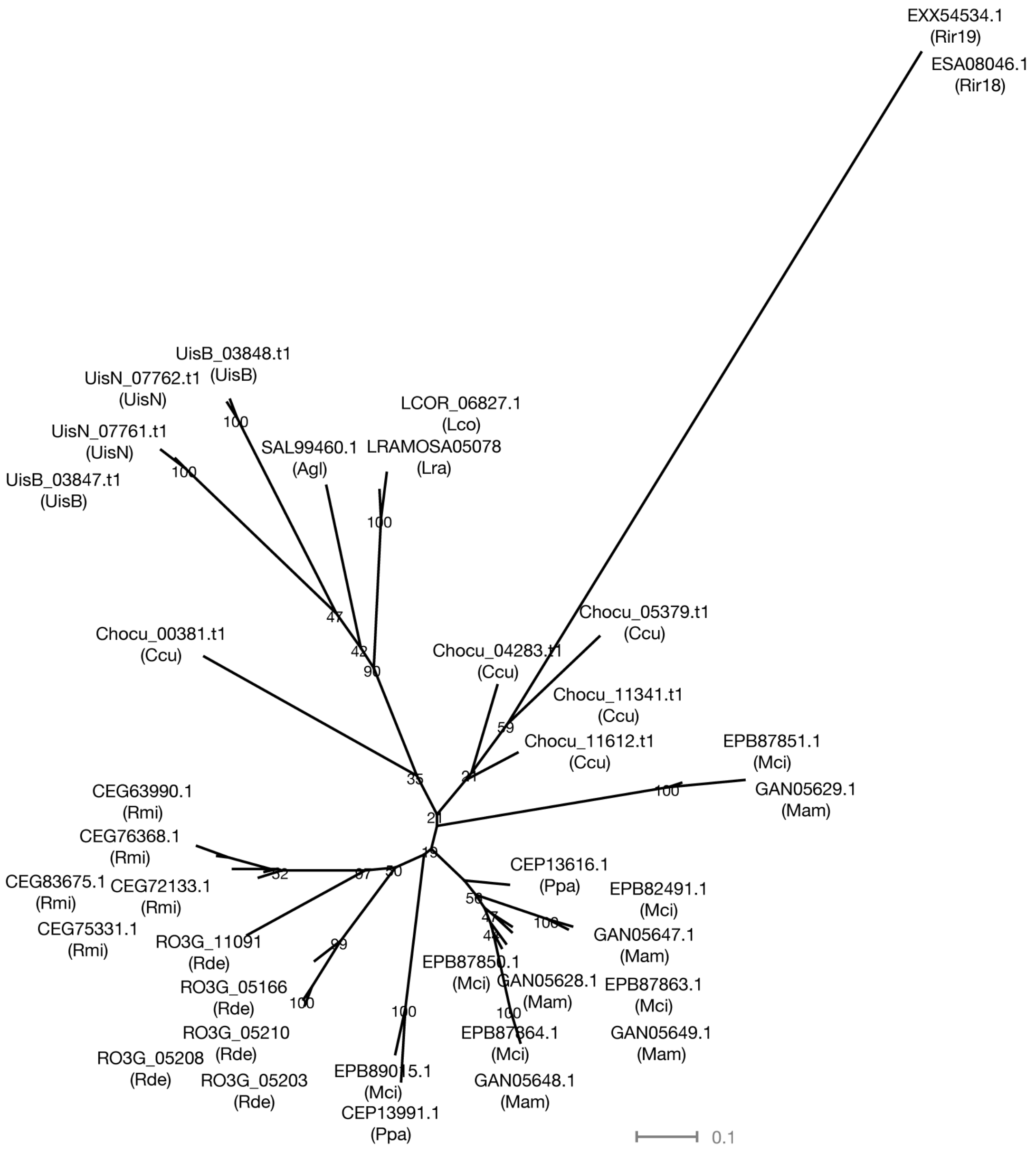

Supplementary Figure S13. Gene tree of *tsp2*. The labels at nodes indicate the GenBank IDs except for *C. cucurbitarum* and two *Umbelopsis* where the locus tags were used. The abbreviations of organisms are shown in parentheses and the corresponding organism names are listed in the methods section. The bootstrap-based branch supports and the scale bar that represents the mean number of amino acid substitutions per site are shown. The tree was built using RAxML 8.2.7 (-f a -x 12345 -p 12345 -# 100 -m PROTGAMMAWAG).

Supplementary Table S1. Summary of comparatives used in this study.

Comparatives	Order	GenBank ID	Ecotype	Number of Genes
Absidia glauca CBS 101.48	Mucorales	GCA_900079185.1	-	14,891
Lichtheimia corymbifera JMRC:FSU:9682	Mucorales	GCA_000723665.1	Human pathogen[1], saprotroph	13,557
Lichtheimia ramosa	Mucorales	GCA_000945115.1	Human pathogen[2]	11,546
Mucor ambiguus NBRC 6742	Mucorales	GCA_000950595.1	-	11,343
Mucor circinelloides f. *circinelloides* 1006PhL	Mucorales	GCA_000401635.1	Human pathogen[3], wound pathogen[4]	12,227
Parasitella parasitica CBS 412.66	Mucorales	GCA_000938895.1	Facultative mycoparasite[5]	13,408
Rhizopus delemar 99-880	Mucorales	GCA_000149305.1	Human pathogen[6]	17,463
Rhizopus microsporus ATCC 62417	Mucorales	GCA_000325505.1	Human pathogen[7], plant pathogen[8]	22,427
Umbelopsis isabellina B7317	Mucorales	GCA_000697415.1	-	9,082
Umbelopsis isabellina NBRC 7884	Mucorales	GCA_000534915.1	-	9,460
Rhizophagus irregularis DAOM 181602	Glomerales	GCA_000439145.2	Mycorrhiza[9]	29,830
Rhizophagus irregularis DAOM 197198w	Glomerales	GCA_000597685.1	Mycorrhiza[9]	29,911
Colletotrichum graminicola M1.001	Glomerellales	*GCA_000149035.1*	Plant pathogen[10]	12,020
Colletotrichum higginsianum IMI 349063	Glomerellales	*GCA_000313795.2*	Plant pathogen[10]	16,141
Ustilago maydis 521	Ustilaginales	*GCA_000328475.2*	Plant pathogen[11]	6,783

References

1. Schwartze, V. U. *et al.* Gene expansion shapes genome architecture in the human pathogen *Lichtheimia corymbifera*: an evolutionary genomics analysis in the ancient terrestrial mucorales (Mucoromycotina). *PLoS Genet.* **10**, e1004496 (2014).
2. Kutlu, M. *et al.* Pulmonary mucormycosis due to *Lichtheimia ramosa* in a patient with HIV infection. *Mycopathologia* **178**, 111-115 (2014).
3. Khan, Z. U., Ahmad, S., Brazda, A. & Chandy, R. *Mucor circinelloides* as a cause of invasive maxillofacial zygomycosis: an emerging dimorphic pathogen with reduced susceptibility to posaconazole. *J. Clin. Microbiol.* **47**, 1244-1248 (2009).
4. Nishijima, K. A., Wall, M. M., Chang, L. C., Wei, Y. & Wong, D. K. W. First Report of Association of *Mucor circinelloides* on Noni (*Morinda citrifolia*) in Hawaii. *Plant Dis.* **95**, 360-360 (2010).
5. Roden, M. M. *et al.* Epidemiology and outcome of zygomycosis: a review of 929 reported cases. *Clin. Infect. Dis.* **41**, 634-653 (2005).
6. Cheng, V. C. *et al.* Outbreak of intestinal infection due to *Rhizopus microsporus*. *J. Clin. Microbiol.* **47**, 2834-2843 (2009).
7. Partida-Martinez, L. P. & Hertweck, C. Pathogenic fungus harbours endosymbiotic bacteria for toxin production. *Nature* **437**, 884-888 (2005).
8. Schultze, K., Schimek, C., Wostemeyer, J. & Burmester, A. Sexuality and parasitism share common regulatory pathways in the fungus *Parasitella parasitica*. *Gene* **348**, 33-44 (2005).
9. Tisserant, E. *et al.* Genome of an arbuscular mycorrhizal fungus provides insight into the oldest plant symbiosis. *Proc. Natl. Acad. Sci. U.S.A.* **110**, 20117-20122 (2013).
10. O'Connell, R. J. *et al.* Lifestyle transitions in plant pathogenic *Colletotrichum* fungi deciphered by genome and transcriptome analyses. *Nat. Genet.* **44**, 1060-1065, (2012).
11. Kamper, J. *et al.* Insights from the genome of the biotrophic fungal plant pathogen *Ustilago maydis*. *Nature* **444**, 97-101 (2006).
